# Phylogenomic and functional analyses of salmon lice aquaporins uncover the molecular diversity of the superfamily in Arthropoda

**DOI:** 10.1186/s12864-015-1814-8

**Published:** 2015-08-19

**Authors:** Jon Anders Stavang, Francois Chauvigné, Heidi Kongshaug, Joan Cerdà, Frank Nilsen, Roderick Nigel Finn

**Affiliations:** Sea Lice Research Centre, Department of Biology, Bergen High Technology Centre, University of Bergen, 5020 Bergen, Norway; Institute of Marine Research, Nordnes, 5817 Bergen, Norway; Institut de Recerca i Tecnologia Agroalimentàries (IRTA)-Institut de Ciències del Mar, Consejo Superior de Investigaciones Científicas (CSIC), 08003 Barcelona, Spain

**Keywords:** Aquaporin, Aquaglyceroporin, Arthropod, Crustacea, Copepod, Salmon louse, Lepeoptheirus, Parasite, Atlantic salmon, Evolution, Osmoregulation, Fluid homeostasis, Permeability, Selectivity

## Abstract

**Background:**

An emerging field in biomedical research is focusing on the roles of aquaporin water channels in parasites that cause debilitating or lethal diseases to their vertebrate hosts. The primary vectorial agents are hematophagous arthropods, including mosquitoes, flies, ticks and lice, however very little is known concerning the functional diversity of aquaporins in non-insect members of the Arthropoda. Here we conducted phylogenomic and functional analyses of aquaporins in the salmon louse, a marine ectoparasitic copepod that feeds on the skin and body fluids of salmonids, and used the primary structures of the isolated channels to uncover the genomic repertoires in Arthropoda.

**Results:**

Genomic screening identified 7 aquaporin paralogs in the louse in contrast to 42 in its host the Atlantic salmon. Phylogenetic inference of the louse nucleotides and proteins in relation to orthologs identified in Chelicerata, Myriapoda, Crustacea and Hexapoda revealed that the arthropod aquaporin superfamily can be classified into three major grades (1) classical aquaporins including Big brain (Bib) and Prip-like (PripL) channels (2) aquaglyceroporins (Glp) and (3) unorthodox aquaporins (Aqp12-like). In Hexapoda, two additional subfamilies exist as Drip and a recently classified entomoglyceroporin (Eglp) group. Cloning and remapping the louse cDNAs to the genomic DNA revealed that they are encoded by 1–7 exons, with two of the Glps being expressed as N-terminal splice variants (Glp1_v1, −1_v2, −3_v1, −3_v2). Heterologous expression of the cRNAs in amphibian oocytes demonstrated that PripL transports water and urea, while Bib does not. Glp1_v1, −2, −3_v1 and −3_v2 each transport water, glycerol and urea, while Glp1_v2 and the Aqp12-like channels were retained intracellularly. Transcript abundance analyses revealed expression of each louse paralog at all developmental stages, except for *glp1_v1*, which is specific to preadult and adult males.

**Conclusions:**

Our data suggest that the aquaporin repertoires of extant arthropods have expanded independently in the different lineages, but can be phylogenetically classified into three major grades as opposed to four present in deuterostome animals. While the aquaporin repertoire of Atlantic salmon represents a 6-fold redundancy compared to the louse, the functional assays reveal that the permeation properties of the different crustacean grades of aquaporin are largely conserved to the vertebrate counterparts.

**Electronic supplementary material:**

The online version of this article (doi:10.1186/s12864-015-1814-8) contains supplementary material, which is available to authorized users.

## Background

Aquaporins are protein channels, which are assembled as tetramers in biological membranes to facilitate the rapid, yet selective conductance of water and other small solutes and gasses down their concentration gradients [1]. Each protomer is comprised of a conserved tertiary structure consisting of 6 transmembrane domains with two hemihelices that typically project opposing Asn-Pro-Ala (NPA) motifs as part of a central proton-excluding selectivity filter [2, 3]. In some channels, the NPA motifs may be modified for other functions [4, 5]. An additional selectivity filter located in the outer channel vestibule typically consists of aromatic amino acids and an Arg residue (ar/R), which determines the molecular substrate specificity of the pore [6, 7]. Previous research has shown that aquaporins are encoded in the genomes of nearly all organisms studied to date, with diverse subfamilies expressed in prokaryotic and eukaryotic organisms [8, 9]. Although high gene copy numbers are found in plants and deuterostome animals, the superfamily has been categorised into four major grades consisting of classical water-selective type aquaporins (Aqp0, −1, −2, −4, −5, -5-like, −6, −14, and −15), Aqp8-type aquaamoniaporins (Aqp8 and −16), aquaglyceroporins (Glp, Aqp3, −7, 9, −10 and −13) and unorthodox aquaporins (Aqp11 and −12) [9–13].

Recent research in the biomedical field has begun to focus on the roles of aquaporin water channels in drug-resistant parasites as potential therapeutic targets [14, 15]. This includes aquaporins expressed in protozoan parasites causing malaria, toxoplasmosis, trypanosomiasis and leishmaniasis, and metazoan parasites causing fascioliasis and schistosomiasis in mammals [14, 16–20]. Experiments have shown that double allelic disruption of the major glycerol-transporting channel (AQP9) in mouse erythrocytes inhibits the virulence of the malaria causing *Plasmodium* species of parasite by attenuating its growth and lipogenesis [17, 18]. Other studies have investigated aquaporins in the hexapod mosquitoes (Diptera) and arachnid ticks (Acari) that act as vectors for such parasites or other bacterial and viral pathogens [21–29]. Unlike the protozoan parasites, which infect endogenous tissues, however, mosquitoes and ticks are ectoparasites, which gorge on the blood of mammals as part of their reproductive cycle [30, 31]. This hematophagous behaviour induces osmoregulatory stress due to the manifold quantities of fluid imbibed [24, 25, 32, 33], and a number of aquaporins have been identified that are considered to be involved in the fluid homeostasis of ingestion, excretion and chemoreception. For dipteran mosquitoes, chironomids and tsetse flies, this includes up to six aquaporins variously labelled as AeaAQP or AQP1 to −6 [21, 22, 25–27, 29, 32, 34], which may be confused with the mammalian aquaporins (AQP1 to −6) encompassing both water-selective channels and Glps. For other insects, an alternative nomenclature has been partially applied [35], based upon their phylogeny and function, including water-selective channels similar to the *Drosophila* intrinsic protein (Drip) [36–40], a water and urea-transporting channel similar to *Pyrocoelia rufa* integral protein (Prip) [41, 42], and a cell-adhesive, cation-permeating neurogenic protein termed big brain (Bib) [43, 44]. The aquaporin nomenclature for ticks is also confusingly annotated as AQP1 or −2 [23, 24, 28], which are Glps rather than the water-selective channels found in mammalian genomes. As for the mouse AQP9 trial, it is one of the tick Glps that has been targeted for vaccine development. In this instance, recombinant regions of the Glp (RmAQP1) were intramuscularly injected into cattle, and provided sufficient antigenic capacity to reduce tick infection by up to 75 % [28].

An equally problematic blood-feeding ectoparasite with broad resistance to available drugs is the saltwater salmon louse (*Lepeophtheirus salmonis*), which is a caligid crustacean copepod (class: Maxillipoda) causing severe infections of salmonids across the Atlantic and Pacific regions [45, 46]. It has been established that in contrast to its salmonid host, which is an osmoregulator, the ionic concentration of the louse’s hemolymph conforms to full strength seawater and remains significantly hyperosmotic in dilute seawater, however, cell volume regulation breaks down in fresh and brackish waters [47]. These features suggest that aquaporins likely play important physiological roles in the louse’s fluid homeostasis, which, given the divergent osmoregulatory capabilities to its salmonid host, could represent eminent therapeutic targets. To date, however, almost nothing is known of the genomic diversity of these channels in crustaceans, which are represented by tens of thousands of species [48]. To redress this lack of knowledge we screened the available genome of the salmon louse, and identified the full complement of water channel genes and cloned the corresponding cDNAs. We then used maximum likelihood and Bayesian inference of the louse nucleotides and deduced proteins to classify the genomic repertoires of aquaporins in the four extant lineages of Arthropoda, the Chelicerata (scorpions, spiders, ticks), Myriapoda (centipedes), Crustacea (water fleas, copepods) and Hexapoda (insects). We subsequently functionally characterised the permeability properties and stage specific-expression patterns of each channel, and used the integrated results to propose a uniform aquaporin nomenclature for arthropods and vertebrates.

## Results and discussion

### The aquaporin superfamily of the salmon louse

**Fig. 1:**
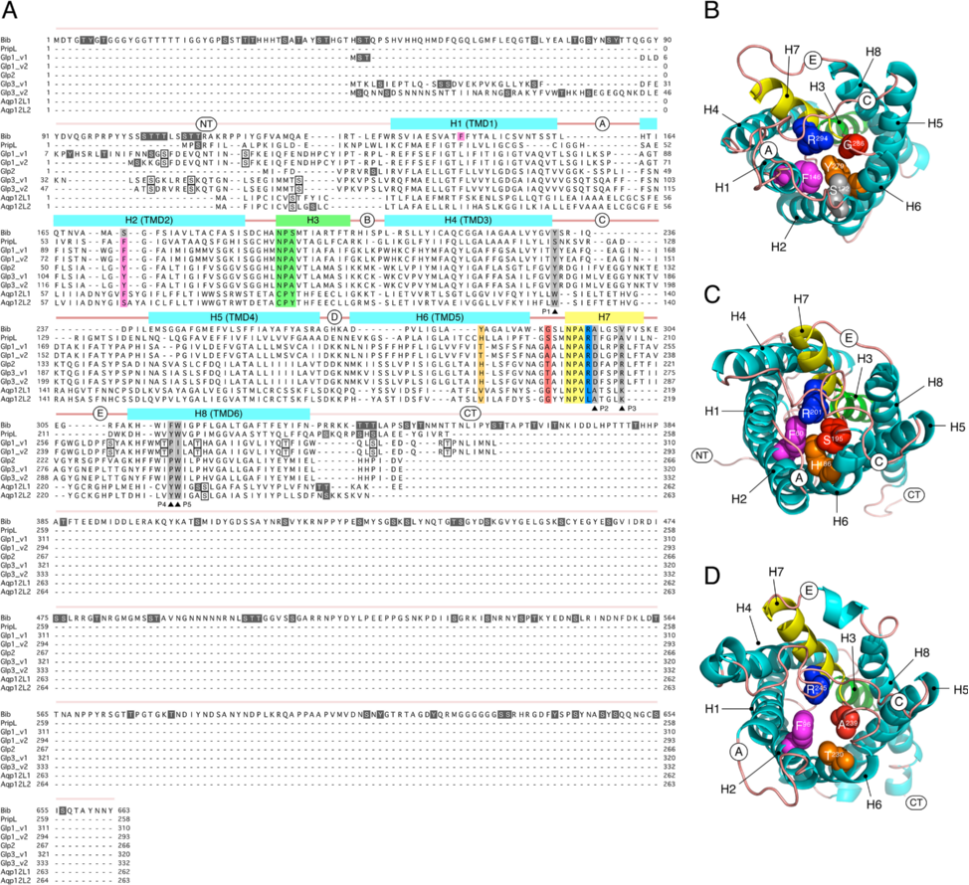
Molecular structures of salmon lice aquaporins. **a** Multiple sequence alignment of the full-length proteins showing secondary structural features including the N-terminal regions (NT), α-helices (H), transmembrane domains (TMD), loops A-E and C-termini (CT). Unique (white text on black background) and common (boxed residues) putative phosphorylation sites are shown for the N- and C-teminal regions. The two Asn-Pro-Ala (NPA) motifs are highlighted in green and yellow on hemihelices 3 and 7, respectively. Aromatic-arginine (ar/R) residues associated with the selectivity filter in the outer channel vestibule are highlighted in magenta, organge, red and blue, respectively. The position of Ser^172^, which normally corresponds to the ar/R residue on H2 is highlighted in gray for Bib. Residues predicted to confer glycerol selectivity (P1–P5) are highlighted in gray [50]. **b**, **c** and **d** Extracelluar views of cartoon renders of Bib, PripL and Glp1_v1 with ar/R residues (spacefill) coloured according to highlighted residues in (A). Helices and extracellular loops are annotated

The screening of the salmon louse genome identified nine putative sequences with homologies to the aquaporin family of proteins. Each putative mRNA sequence was isolated and cloned using gene-specific primers and rapid amplification of cDNA ends (RACE) analyses. This resulted in 9 cDNAs with open reading frames ranging in length from 777–1992 bp, encoding proteins of 258–663 amino acids, which, following phylogenetic analyses (see below), we termed Bib, Prip-like (PripL), Glp1-3, Aqp12-like1 (Aqp12L1) and Aqp12-like 2 (Aqp12L2) (Fig. 1a). We found no evidence for the existence of a putative lsal-evj-541-119 Aqp (BT120567) deposited in GenBank, either experimentally using PCR with gene specific primers or *in silico* via searches of the salmon louse genome.

Alignment and *in silico* analyses of the secondary and tertiary structures showed that each louse protein consists of six transmembrane domains, two Asn-Pro-Ala (NPA)-like motifs at the termini of two hemihelices, and five loops A – E typically found in mammalian aquaporins [49]. The largest protein, which has a deduced length of 633 amino acids and a predicted molecular mass of 72 kD is Bib, and harbours a long N-terminus (135 amino acids) and an exceptionally long C-terminus (329 amino acids), similar to the subdomain structure of the *Drosphila* Bib molecule [43]. The N-terminus of the louse Bib is thus equivalent in length to the membrane-spanning domains (169 amino acids), while the C-terminus is twice the size. Three dimensional reconstruction of the louse Bib revealed that in contrast to the majority of aquaporins displaying an ar/R residue on transmembrane domain 2 (TMD2), the putative constriction residue is Phe^146^ on TMD1, rather than Ser^172^ on TMD2 (Fig. 1b). Conversely, PripL, which represents the smallest aquaporin in the louse superfamily with 258 amino acids and a predicted molecular mass of 27.8 kD, displays the more canonical ar/R constriction and functional (P1–P5) residues associated with the water-selective branch of aquaporins (Fig. 1a, c) [7, 50]. The tertiary structures of the Glps, which range in length from 266–332 amino acids with predicted molecular masses from 28.6–35.8 kD, appear less constricted in the central pore due to a generally more relaxed fold conformation and an uncharged Thr on TMD5 of Glp1_v1 and −1_v2 (Fig. 1d, Additional file 1: Figure S1A). Interestingly, although Glp2, −3_v1 and −3_v2 harbour a His on TMD5 that is typical of water-selective channels [7], the *in silico* models indicate that the TMD5 His does not strongly protrude into the central channel as in PripL and thus may not present a hindrance to glycerol conductance (Additional file 1: Figure S1B-D). The remaining sequences, Aqp12L1 and -12L2, were more divergent (11–18 % identity over the membrane-spanning domains compared to the other louse aquaporins) with lengths of 262–263 amino acids and predicted molecular masses of 29.1 and 28.8 kD, respectively. These latter channels have an ungapped alignment identity of 61.8 % and encode non-canonical CPY and NPV motifs associated with unorthodox members of the aquaporin superfamily [4]. Three-dimensional models based upon the structure mask of AqpZ (2ABM, Z-score = 33–34) indicate that Aqp12L1 is highly constricted due to a Phe on TMD2, while Aqp12L2 has a more open conformation due to the substitution of the Phe for an Ile in this position (Additional file 1: Figure S1E-F).

### Molecular phylogeny of salmon louse aquaporins uncovers the genomic repertoires in Arthropoda

**Fig. 2:**
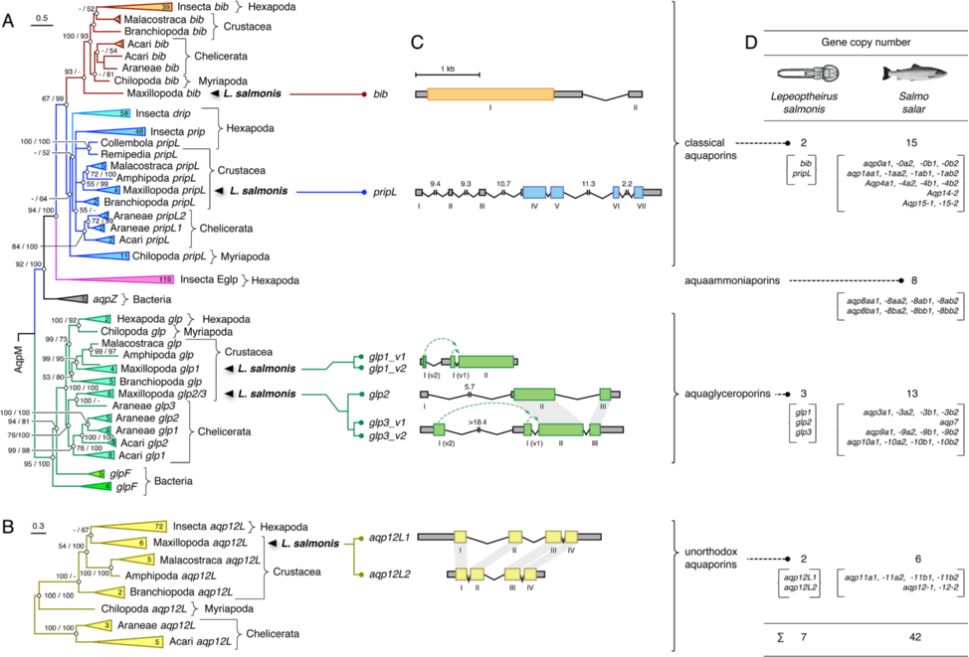
Phylogeny of the genomic repertoires of arthropod aquaporins compared to the tetraploid complement in Atlantic salmon. **a** Maximum likelihood of arthropod orthodox aquaporins. The tree is rooted with AqpM and was inferred from 30 million MCMC generations on 484,924 nucleotide sites from a codon alignment of 350 non-redundant aquaporins. Posterior probabilities of the codon/amino acid analyses are shown at each node, with “-“ representing a polytomy (<50 %). The numbers of taxa are indicated in the collapsed branch triangles. Scale bars indicate the rate of nucleotide substitution per site. **b** Bayesian majority-rule phylogeny of arthropod unorthodox aquaporins. The tree is mid-point rooted and was inferred from 5 million MCMC generations on 87,613 nucleotide sites in a codon alignment of 94 non-redundant aquaporins. Annotations as in (**a**). **c** Genomic organisation of the salmon louse aquaporins. Colored and gray boxes indicate coding and non-coding exons, respectively. Long introns are annotated in kb, and gray backgrounds linking paralogs highlight conserved exons. Exon numbers are annotated with roman numerals, with the first proximal and distal exons of splice variants labelled A and B, respectively. **d** Gene copy numbers and orthology of salmon louse and Atlantic salmon aquaporins

To understand the evolutionary history of the salmon louse aquaporins, each paralog was subjected to phylogenetic analyses in relation to 435 orthologs retrieved from Chelicerata, Myriapoda, Crustacea and Hexapoda using maximum likelihood and Bayesian protocols. In addition, 21 prokaryotic AqpZ, GlpF and AqpM orthologs were included to ensure that none of the isolated louse aquaporins were derived from prokaryotic endosymbionts. Two main arthropod data sets were constructed consisting of 350 non-redundant orthodox aquaporins (Fig. 2a) and 94 non-redundant unorthodox aquaporins (Fig. 2b). These analyses revealed that two of the louse paralogs, Bib and PripL are members of the classical water-selective branch of aquaporins as judged by their derivative clustering above bacterial AqpZ. Although the salmon louse Bib clustered as an arthropod Bib, it did not cluster together with the other malacostracan (crab and crayfish) or branchiopod (water flea) crustaceans. This is primarily due to the derived nature of the louse sequence, which has a membrane-spanning identity of 27–28 % compared to the 46 other arthropod Bibs analysed. In contrast the water flea (*Daphnia pulex*) Bib is less derived with 32–38 % identity to the membrane-spanning regions of other arthropod Bib-like proteins, and clusters as a sister branch to Hexapoda consistent with the pancrustacean model of arthropod evolution [51, 52]. The present phylogenetic data thus reveal that neurogenic Bib channels are not only found in dipteran or hemipteran insects [25, 32, 43, 53–55], but are encoded in the genomes of all extant lineages of Arthropoda.

The second water-selective type classical aquaporin isolated from the louse clustered together with other maxillipod and crustacean water channels that are closely related to insect Prips. Previously Prip-like channels, variously named as Prip, AQP, AQP1, AQP2, AQP4A, AQP4B have only been identified in Hexapoda [25–27, 32, 34, 35, 41, 42, 55–58], however the present findings reveal that Prip-like water channels exist throughout the Arthropoda. For most species, including the salmon louse, single copy genes were identified, although spiders (Araneae) have at least two, and the centipede species studied has specifically expanded this class of aquaporins with 11 divergent paralogs (27–28 % identity). Very recently we have also identified multiple *prip*-like genes in the oldest hexapod lineages, including Archeognatha, Diplura, Collembola and Protura [59]. In addition to the *prip* genes, the phylogenetic analyses show that two insect-specific classes of aquaporin exist as *drip* and the recently identified glycerol transporters termed entomoglyceroporins (*eglps* [59]). We therefore conclude that arthropods have two ubiquitous classes of classical aquaporin proteins termed Bib and PripL, while while hexapods have four, Bib, Prip, Drip and the glycerol-transporting Eglp group.

The remaining orthodox aquaporins isolated from the salmon louse clustered as canonical *glps*, as opposed to orthologs in bacterial symbionts (Fig. 2a). Interestingly the phylogenetic signal revealed that *glp* evolution in arthropods appears to be polyphyletic, with multiple gene copies existing in the different lineages. For the louse, two subclusters are observed, with the first including *glp1* co-clustering with other crustacean glps, while the second, which includes *glp2* and −*3*, clusters with chelicerate *glps*. These data suggest that *glp1*-type genes share a common ancestor in Crustacea, while *glp2*- and −*3*-type genes have a different origin. In contrast to the polyphyletic evolution of maxillipod and chelicerate *glps*, however, the data for Branchiopoda indicate that the *glp* repertoires specifically expanded within this lineage with 6 gene products on the same branch.

The present data reveal that *aqp12*-related genes are encoded in the genomes of each of the major lineages of arthropod (Fig. 2b). This latter finding is therefore consistent with previous studies that identified Aqp12-like orthologs in Diptera [32, 60], Tardigrada (water bears) and Cnidaria (corals and sea anemones), the latter of which represent basal members of the Panarthropoda and Metazoa, respectively [9, 61]. Unlike the other arthropod species studied here, however, we show that the salmon louse encodes two unorthodox aquaporins (Aqp12L1 and -12L2), which co-cluster with other maxillipod Aqp12-like sequences as a sister branch to hexapods. Consequently, despite the relatively low amino acid conservation between the Aqp12L1 and -12L2 paralogs (61.8 %), the phylogenetic data imply that the genes may have diverged within parasitic caligid copepods.

### Genomic structure and N-terminal splice variants

To decipher the genomic organization of the salmon louse aquaporins, we mapped each mRNA sequence, including the 5′ and 3′untranslated regions (UTR), to the genomic DNA. This revealed that Although the *bib* transcript includes a second exon for the 3′UTR, the Bib protein is encoded within a single exon, while the coding regions of all other paralogs are located within 3–4 exons (Fig. 2c). The single coding exon structure of the Bib protein is unusual compared to other arthropod orthologs, which are encoded by five exons in *Drosophila*, up to nine exons in the assassin bug (*Rhodnius prolixus*), and based upon the tblastn searches of whole genome shotgun sequences at least three exons in the common house spider (*Parasteatoda tepidariorum*) and at least five exons in the coastal European centipede (*Strigamia maritima*). The intronless structure of the coding region of the louse *bib* gene thus appears to be specific to the salmon louse, and may have evolved via reverse transcription and replacement of an older split gene as suggested for human G-protein-coupled receptors [62].

The *pripL* gene is by far the longest spanning >43 kb with four coding exons and three non-coding exons in the 5′ UTR. Unlike the prip of the malaria mosquito (*Anopheles gambiae*), which differentially expresses two splice variants in the ovary and gut [27], no splice variants were detected for the louse *pripL*. By contrast two of the louse *glps* (*glp1* and −*3*), which are encoded by 2–3 exons are transcribed as splice variants. In both cases the variation occurs at the N-terminus whereby either a proximal exon (v1) or a distal exon (v2) is spliced. We therefore named these isoforms *glp1_v1*, *glp1_v2* and *glp3_v1*, *glp3_v2*, respectively (Fig. 2c). Interestingly, a *glp1_v1-*like transcript described here for the salmon louse has recently been identified through high-throughput transcriptome sequencing as a sex-specific variant termed AQP3 in a different parasitic copepod, *Caligus rogercresseyi* [63]. To determine whether related splice variants exist in other copepods, we analyzed the available transcriptomes of five species, including the salmon louse, and found that N-terminal splice variation of both *glp1* and −*3*-type genes appears to be a conserved feature within the maxillopod class of Copepoda (see Additional file 1: Figure S2).

Genomic analysis of the *glp2* gene revealed that it is closely related to *glp3* with 77 % nucleotide identity for the overlapping regions of exons II and III. Since this gene has not previously been reported for any organism, and *glp3*-type genes are found in the transcriptome shotgun assemblies of other species of copepod, it seems likely that *glp2* may have recently evolved as a duplicate of *glp3* in the salmon louse. By contrast both of the louse *aqp12*-like genes are expressed in the transcriptomes of other caligid parasitic copepods, such as *Caligus rogercresseyi*, but apparently not in other members of the Crustacea. The genomic organisation of the louse *aqp12L1* and *-12L2* paralogs is conserved with 4 exons with an extended first intron in the *aqp12L1* gene (Fig. 2c). Although the *Drosphila aqp12*-like gene is also split into 4 exons, this is not a conserved feature of unorthodox aquaporins in Arthropoda as the number of exons varies between species with up to 8 exons in butterflies (Lepidoptera).

### Aquaporin orthology between parasite and host

To identify potential therapeutic targets, we compared the seven paralogs isolated from the louse to the 42 orthologs recently identified in its tetraploid host, the Atlantic salmon [9, 64–67], we aligned the two superfamilies and examined their phylogenetic distributions using Bayesian inference. These results provided robust statistical evidence that the louse *bib* and *pripL* gene products are closely related to the salmon *aqp4* paralogs, and thus members of the classical grade of aquaporins (Additional file 1: Figure S3). Similarly the louse *glp* and *aqp12*-like gene products are related to the salmon aquaglyceroporins and unorthodox aquaporins, respectively, revealing that the salmon louse superfamily consists of 3 major grades of aquaporins, while that of the Atlantic salmon consists of four. Consequently due to serial rounds of whole genome duplication in tetraploid Salmonidae [68, 69], there is a 6-fold redundancy of aquaporins encoded in the genome of Atlantic salmon compared to the salmon louse. A summary of these findings is given in Fig. 2d which illustrates the absence of vertebrate *aqp8*-type aquaammoniaporins in the louse. Based upon the broader molecular phylogenetic analysis (Fig. 2a), the present data further indicate that this is also true for each of the major lineages of arthropod.

### Channel permeation properties of the salmon louse aquaporins

**Fig. 3:**
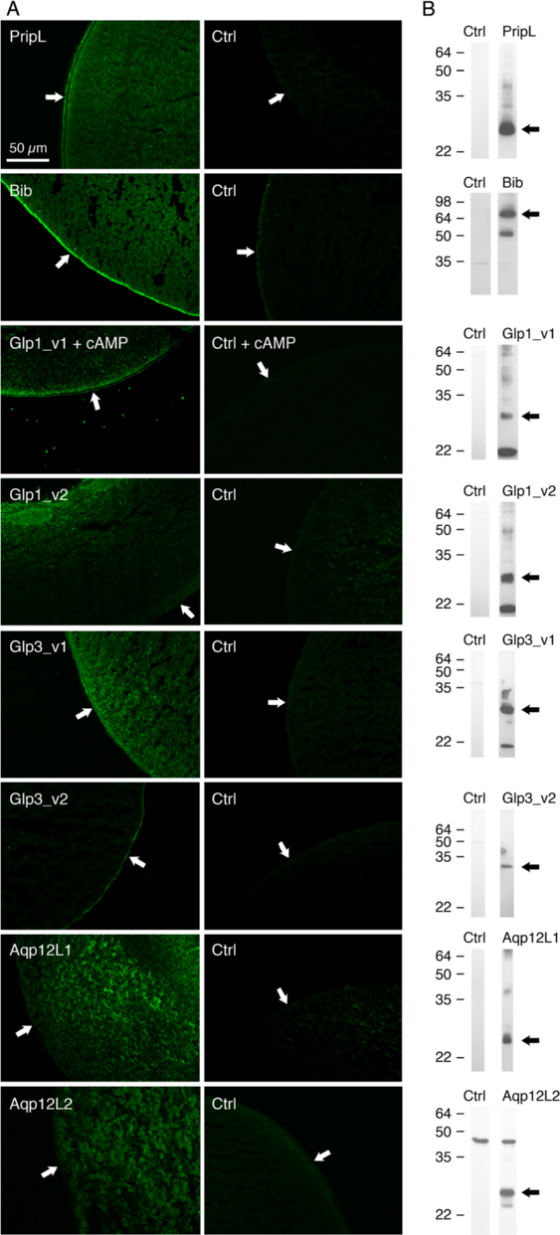
Immunolocalisation of salmon louse aquaporins expressed in *X. laevis* oocytes. **a** Immunofluorescence photomicrographs of paraffin sections of water- (Ctrl) and salmon louse cRNA-injected oocytes probed with paralog-specific antisera followed by Cy3-labeled anti-rabbit IgG. Arrows point to the plasma membrane. Sections shown are representative of five different sections per treatment showing identical staining. **b** Representative immunoblots of total oocyte membranes (0.5-3 oocyte equivalents per lane) with molecular markers shown to the left. Arrows point to bands that match the predicted molecular mass of each aquaporin paralog, while lower bands likely repesent misfolded and degraded proteins. For Aqp12L2 a cross-reaction is seen against a ~45 kD *X. laevis* protein

**Fig. 4:**
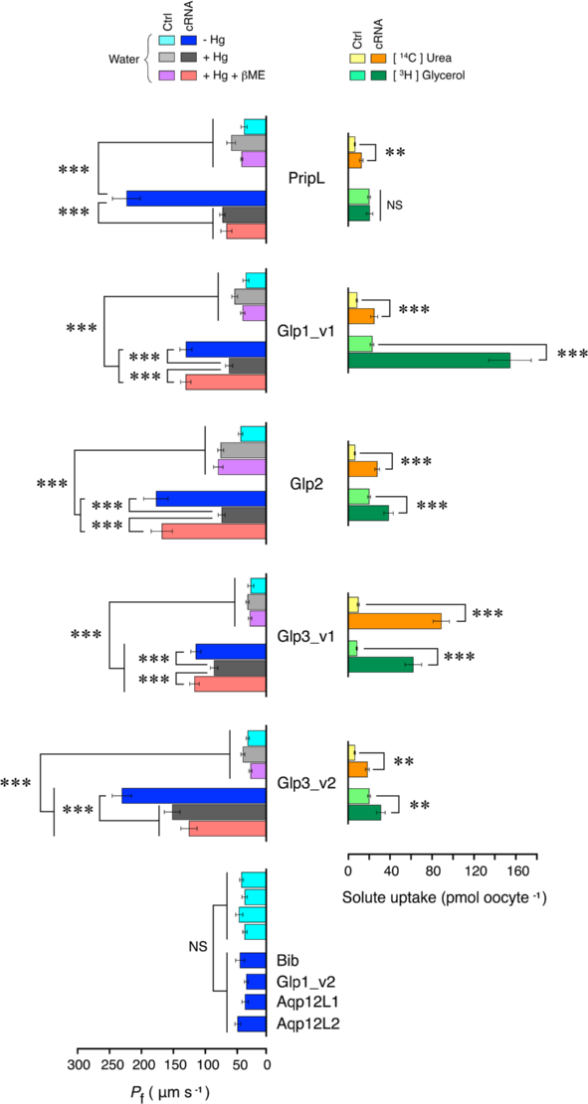
Functional characterization of salmon louse aquaporins. Osmotic water permeability in the presence and absence of HgCl_2_ and βME (*P*_f_, left panel), and urea and glycerol uptake (right panel) of *X. laevis* oocytes expressing the salmon louse aquaporins. Values (mean ± SEM; n = 10–15 oocytes per treatment) are from representative experiments. Statistical differences (* = p < 0.05, ** = p < 0.01, ***p < 0.001) are indicated with brackets

To corroborate the phylogenetic and structural analyses, the functional permeation properties of the salmon louse aquaporins were examined by heterologous expression in *Xenopus laevis* oocytes followed by measurement of the oocyte osmotic water permeability (*P*_f_) in hypotonic media or uptake of radioactive solutes (glycerol and urea). The subcellular localization of the aquaporins in the injected oocytes was monitored by immunofluorescence microscopy and immunoblotting using specific affinity-purified antibodies raised in rabbits against synthetic C-terminal peptides (see Additional file 1: Table S1). Injection of 5–10 ng of cRNA and subsequent expression of PripL, Glp3_v1 and Glp3_v2 targeted the proteins to the oocyte plasma membrane (Fig. 3) and induced a 2.5–4.5-fold increase in the oocyte water permeability compared to water injected controls (Fig. 4). Although antibody development for the Glp2 paralog was not successful, the heterologous expression experiments showed that it functions as a water transporter. By contrast, oocytes expressing high cRNA titres (20 ng) of the Glp1_v1 splice variant were not permeable to water due to the retention of the channel just below the oocyte plasma membrane (see Additional file 1: Figure S4). Addition of dibutyryl cyclic adenosine monophosphate (cAMP) in the bathing media was necessary to traffic the channel to the plasma membrane (Fig. 3) and induce the increase in water permeability (Fig. 4). This suggested that a cAMP-dependent phosphorylation mechanism may be involved in the trafficking of the Glp_v1 channel to the plasma membrane as reported for some vertebrate [10, 12, 70–74] and plant aquaporins [75–77]. An *in silico* analysis of the putative phosphorylation sites indicates that five residues in the N-terminus of Glp1_v1 (Ser^2^, Thr^3^, Tyr^9^, Thr^14^and Ser^21^) differ in phosphorylation potential compared to the Glp1_v2 isoform (Fig. 1a), implying that a casein type kinase, a protein kinase type C or a receptor tyrosine kinase [78] may be involved in the trafficking of this splice variant to the oocyte plasma membrane. By contrast, the 17 amino acid shorter Glp1_v2 isoform, which retains only one unique putative phosphorylation site, was not permeable to water in *Xenopus* oocytes, even with cAMP added to the media. The immunolocalization and Western blot experiments revealed that Glp1_v2 was retained intracellularly (Fig. 3a) and was quickly degraded in the oocytes (see ~20 kD band in Fig. 3b).

While cAMP-dependent membrane trafficking involving the phosphorylation of C-terminal aquaporin residues is well established [10, 12, 70–77], the role of the N-terminus in this pathway is less common. Nevertheless, the N-terminus has been associated with trafficking of some mammalian channels, including AQP4 and AQP6 [79, 80]. In the present context our data suggest that only the N-terminus of the Glp1 isoforms retains putative phosphorylation sites which may be the targets of kinases in the cAMP-dependent trafficking mechanism. Further research will be necessary to decipher the precise mechanism involved.

Addition of 0.3 mM HgCl_2_ abolished the water transport function of the PripL, Glp1_v1 and −2 channels and significantly (*p* < 0.001) inhibited the water permeability of oocytes expressing Glp3_v1 and −3_v2 (Fig. 4). This inhibition was reversed by the addition of β-mercaptoethanol (βME) to Glp1_v1, −2 and −3_v1, but not to PripL or Glp3_v2. Studies of mammalian AQP1 channels have established that the mercury-induced inhibition is associated with the binding of Hg^2+^ to a Cys^189^ residue upstream of the second NPA motif resulting in a collapse in the orientation of several residues at the ar/R region [81–83]. Consequently, the irreversible mercury sensitivity of the louse PripL channel may therefore be associated with three Cys residues (Cys^77^, Cys^184^ and Cys^185^) located upstream upstream of the two NPA motifs (see Fig. 1a). However, as for certain piscine [11], mammalian [84, 85] and plant [86–88] aquaporins, the mercury sensitivity of the other louse paralogs was independent of any Cys residue upstream of either NPA motif. This observation reaffirms that the mechanism of mercurial inhibition is complex and likely paralog-specific due to the position of the Cys residues in the primary structures [11, 85, 88].

Heterologous expression of the Bib, Aqp12L1 and -12L2 channels in *X. laevis* oocytes did not induce any significant swelling compared to the water injected controls (Fig. 4). The immunolocalization and Western blot experiments revealed that despite being translated and expressed as 27–29 kD proteins, both of the unorthodox aquaporins were retained intracellularly (Fig. 3a, b). Interestingly, although a cross reaction was noted for the Aqp12L1 antibody in the Western blot, it was not observed in the control immunohistochemical analysis, indicating that it is the Aqp12L2 protein located in the yolk vesicles of the cRNA-injected oocytes. In contrast to these observations, the affinity-purified antibody developed against a C-terminal peptide of Bib revealed that it is expressed as a 72 kD protein and trafficked to the oocyte plasma membrane (Fig. 3a, b). Consequently the absence of water transport through the louse Bib paralog is potentially due to the unusual constriction residue (Phe^146^) on TMD1 (Fig. 1b), and may support its role as an ion channel and/or a cell adhesion molecule rather than a water transporter as reported for the *Drosophila* ortholog [44, 89]. Further research will be necessary to identify the molecular and cell physiological function of the louse Bib.

The uptake assays for urea and glycerol revealed that PripL transports a significant amount of urea compared to the controls, but not glycerol, while all of the Glps expressed in the plasma membrane transport both urea and glycerol (Fig. 4). These findings are consistent with the open pore configurations of the Glps (Fig. 1d; Additional file 1: Figure S1), and a recent report demonstrating that a Prip ortholog isolated from the German cockroach (*Blattella germanica*) transports water and urea, but not glycerol [42].

### Expression analysis of salmon louse aquaporins

**Fig. 5:**
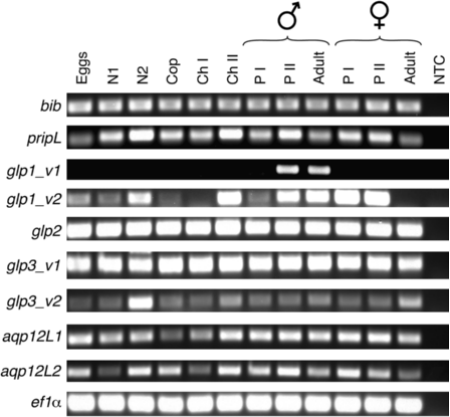
Developmental expression of salmon louse aquaporins. RT-PCR data are shown against expression of elongation factor 1α for egg, nauplius (N), the infective copepodite (Cop) stage and the obligate chalimus (Ch) pre-adult (P and adult parasitic stages for males and females. No template controls (NTC) were included to exclude the possibility of non-specific amplification due to primer-dimer formation or contamination

To gain insight into the developmental importance of the louse aquaporins, the stage-specific expression patterns were evaluated in whole individuals in relation to elongation factor 1α (*ef1α*) by RT-PCR. With the exception of *glp1_v1*, each paralog was detected in all developmental stages from egg to adult (Fig. 5). The most striking expression pattern was observed for the *glp1_v1* isoform, which is expressed in pre-adult II and adult males, while the *glp1_v2* isoform is expressed in the pre-adult and adult stages of both sexes. This finding thus supports the recent observation of the differential expression of an *aqp3*-annotated gene in the parasitic copepod *C. rogercresseyi* [63]. It is not clear, however, whether *C. rogercresseyi* also expresses a shorter splice variant of this gene. To date, very few arthropod aquaporins have been identified as splice variants, and sex-specific splicing has until now only been reported in the malaria mosquito *Anopheles gambiae* [27]. The present study reveals that the functional repertoire of crustacean aquaporins is increased through splice-variation, which can contribute to the sex-specific fluid homeostasis of crustaceans. Further studies will be necessary to understand how the aquaporin genes are regulated and what are the subcellular sites of expression, in order to better understand the differential roles of the parasite channels compared those in the host.

## Conclusions

In the present study we identified seven aquaporin genes in the salmon louse compared to 42 in its host, the Atlantic salmon. However, in contrast to the salmon repertoire, which is phylogenetically divided into four major grades of classical-type water channels, Aqp8-type aquaamoniaporins, aquaglyceroporins and unorthodox aquaporins, Bayesian inference revealed that the salmon louse and other members of the Arthropoda have lost the Aqp8-type orthologs. The remaining classes of louse and non-hexapod aquaporins are classified as Bib, Prip-like, Glps and Aqp12-like channels, while hexapod insects have an additional Drip channel and a specific group of Eglps, which supplanted the ancestral Glps as the major vehicles of glycerol conductance. Heterologous expression of the cloned louse aquaporins in *X. laevis* oocytes showed that the Prip-like channel is a water and urea transporter, while Bib is not, and all but one of the Glps transports water, glycerol and urea. Expression data showed that each aquaporin is expressed throughout the life cycle of the salmon louse, with one exception where an N-terminal splice variant of Glp1_v1 is specific to pre-adult and adult males. Taken together, these data are the first to reveal the substrate permeability properties of aquaporins in a crustacean organism and uncover the broader genomic diversity of the superfamily in the four extant lineages of Arthropoda.

## Methods

### Genome analysis and cDNA cloning

Salmon louse aquaporin-coding sequences were identified by a basic local alignment search tool (BLAST) in the salmon louse genomic database (sealouse.imr.no). Based on these sequences, we designed gene-specific primers to amplify the 3′- and 5′-terminus by rapid amplification of cDNA ends (RACE) following the manufacturers instructions (SMARTer ^TM^ RACE, Clontech). All experimental procedures were done in accordance with national legislation. RNA was extracted with Trizol (Life Technologies) from laboratory-reared strains of salmon lice (copepodites and adult females), stored on RNA-later^®^ (Life Technologies) and was DNAse treated (Ambion) prior to preparation of 5′and 3′ RACE-cDNA. After sequencing the 5′ and 3′ ends of each aquaporin paralog, new primers with restriction enzyme sites were respectively designed in the 5′UTR and 3′UTR regions in order to produce full length clones that included the open reading frames (ORF). cDNA template was prepared from DNAse treated RNA as described above with the Affinityscript cDNA synthesis kit (Agilent Technologies) followed by a ten-fold dillution of the cDNA. The PCR reaction for producing the aquaporin-clones was carried out using a high fidelity polymerase (Phusion, New England Biosystems, Canada). The resulting PCR product was run on a 1 % agarose gel, purified with a gel extraction kit (Quiagen gel extraction kit), digested with EcoRV/SpeI estriction enzymes and ligated into the *X. laevis* oocyte expression vector pT7Ts – vector [90] using a T4 ligase (Quick ligation kit, New England Biosystems). Finally, the pT7Ts vector containing the ligated aquaporin paralogue was cloned into *E. coli* (Invitrogen, USA), and sequenced plasmids from overnight cultures from single bacterial clones were used in later experiments. The cloned aquaporin mRNA sequences were further used to identify the intron-exon borders of the respective genes by alignment to the genomic sequences of the salmon louse. The experiments were performed in accordance with Norwegian legislation and approved by the Norwegian Animal Research Authority (permits nr. 2010/ 245410 and 2009/186329).

### Phylogenetic and structural analyses

Phylogenetic analyses were performed as described previously [9, 91–93] using maximum likelihood (PAUP v4b10-x86-macosx) and Bayesian inference (Mr Bayes v3.2.2) of codon and deduced amino acid alignments of 435 non-redundant arthropod aquaporins. Orthologous sequences were obtained from open sources (Ensembl, Genbank, whole genome shotgun contigs, transcriptome shotgun assemblies, and expressed sequence tag databases) and assembled as described previously [9]. A full list of the accession numbers of sequences used in the study is provided in Additional file 1: Table S2. Multiple sequence alignments were constructed using default t-coffee v9.01 [94] or L-INS-I MAFFT v7.058b [95] algorithms, and Bayesian model parameters were nucmodel = 4by4, nst = 2, rates = gamma for codon alignments, and aamodel = mixed for amino acid alignments. Markov chain Monte Carlo (MCMC) algorithms were run with 3 heated and 1 cold chain with resulting probability distributions examined for convergence using Tracer version 1.6 (tree.bio.ed.ac.uk/software/tracer/), and majority rule consensus trees summarized with a burnin of 25 %. All trees generated were processed with Archaeopteryx [96] and rendered with Geneious (Biomatters Ltd, New Zealand). A nexus alignment and tree file is deposited in the Dryad Digital Repository. Doi:10.5061/dryad.kd742.

The three-dimensional structures of the salmon louse aquaporins were built using the model leverage option in the Modeller server (modbase.compbio.ucsf.edu). The best scoring models were selected using the slow (Seq-Prf, PSI-BLAST) assignment method and rendered with MacPymol (pymol.org).

### Functional expression in *X. laevis* oocytes

Capped RNAs (cRNAs) were synthesized *in vitro* with T7 RNA Polymerase (Roche) from XbaI-linearized pT7Ts vector containing the different aquaporin sequences. The isolation and microinjection of stage V-VI oocytes was performed as described previously [90]. Oocytes were injected with 50 nl of water alone (control) or containing 1–25 ng cRNA and incubated in Barth’s culture medium (MBS; 0.33 mM Ca(NO_3_)_2_, 0.4 mM CaCl_2_, 88 mM NaCl, 1 mM KCl, 2.4 mM NaHCO_3_, 10 mM Hepes, 0.82 mM MgSO_4_, pH 7.5) at 18 °C over night. The following day the oocytes were de-folliculated and the third day the oocytes were tested for osmotic permeability in a swelling assay.

### Swelling assays

The *P*_f_ was measured in control and injected oocytes from the time course of oocyte swelling in a standard assay. Oocytes were transferred from 200 mOsm MBS to 20 mOsm MBS at room temperature. Oocyte swelling was followed by video microscopy using serial images at 2 s intervals during the first 20 s period. The *P*_f_ values were calculated taking into account the time-course changes in relative oocyte volume [d(V/Vo)/ dt], the molar volume of water (Vw = 18 cm^3^/ml) and the oocyte surface area (S) using the formula Vo [d(V/ Vo )/dt]/[SVw (Osmin - Osmout )]. To examine the inhibitory effect of mercury on *P*_f_ oocytes were pre-incubated for 15 min in MBS containing 0.3 mM HgCl_2_ before and during the swelling assays. To determine the reversibility of the inhibition, the oocytes were rinsed 3 times with fresh MBS and incubated for another 15 min with 5 mM β-mercaptoethanol before being subjected to swelling assays. In some experiments, the cell-permeable dibutyryl cAMP analog (N^6^,2′-O-Dibutyryladenosine 3′,5′-cyclic monophosphate sodium salt; Sigma-Aldrich) was added to the MBS for 30 min prior to and during the hypotonic shock.

### Radioactive solute uptake assays

To determine the uptake of [^3^H]-glycerol (60 Ci/mmol) and [^14^C]-urea (52 mCi/mmol) groups of 6 oocytes, control oocytes or oocytes injected with cRNA, were incubated in 200 μl of MBS containing 20 μCi of the radiolabeled solute (cold solute was added to give 1 mM final concentration) at room temperature. After 10 min (including zero time for subtraction of the signal from externally bound solute), oocytes were washed rapidly in ice-cold MBS three times, and individual oocytes were dissolved in 5 % SDS for scintillation counting.

### Statistical analyses of *P*_f_ and solute uptake

The *P*_f_ and solute uptake data are expressed as mean ± SEM. Data shown are from a representative experiment out of 2–3 different trials (n = 10–15 oocytes per treatment). The measured values were analysed by one-way analysis of variance (ANOVA), after log-transformation of the data when necessary, followed by Tukey‘s pairwise comparison with a 95 % confidence interval (*p* < 0.05).

### Antiobody production

N-terminal or C-terminal peptide sequences of each aquaporin-paralog (see Additional file 1: Table S1) were injected in rabbits to raise paralog specific polyclonal antibodies (Agrisera AB, Sweden). The antisera were affinity-purified against the synthetic peptides as described previously [92], and their specificity confirmed by ELISA, as well as by immunofluorescence microscopy and immunoblotting on *X. laevis* oocytes (see below).

### Immunohistochemistry and immunoblot analysis

For immunohistochemistry, oocytes were fixed for 6 h in 4 % formaldehyde (4 % PFA dissolved in PBS), washed in PBS before being dehydrated in steps by ascending concentrations of ethanol (50, 70, 96 100 % ethanol in H_2_O) and finally in xylene, before being embedded in paraffin. The paraffin embedded oocytes were cut in 7 μm thick sections and the slides de-paraffinised in xylene and rehydrated in a series of descending ethanol baths. Epitope retrieval was done by either a 10 min incubation in 0.1 % Triton X-100, or a 10 min incubation in acetone at −20 °C, followed by three washes with 95 °C citrate buffer (10 mM sodium citrate, pH 6.0). Sections were blocked with 5 % goat serum and 0.1 % BSA for 20 min before the sections were incubated with the paralog-specific antibodies (1:400 dilution). The primary antibodies were detected using FITC anti-rabbit secondary antibodies (1:800 dilution, Sigma-Aldrich). Sections were mounted with Fluoromount aqueous mounting medium (Sigma-Aldrich), and immunofluorescence was observed and documented with a Zeiss imager.z1 microscope (Carl Zeiss MicroImaging, S.L.).

For Western blot analyses, aquaporin-injected oocytes were homogenized in HbA-buffer (20 mM Tris, pH 7.4, 5 mM MgCl_2_, 5 mM NaH_2_PO_4_, 1 mM EDTA, 80 mM sucrose, and protease inhibitor cocktail [Mini EDTA-free; Roche], 20 μL/oocyte), followed by two centrifugations for 5 min at 500 *g* where the supernatant was taken. Finally, a spin for 20 min at maximum speed pelleted the total membranes and this pellet was dissolved in 1x Laemmli buffer (5 μl per oocyte) and heated 10 min at 95 °C. The total membrane extract was loaded on a 12 % SDS gel and run at 90 V for 1.5 h. The separated peptides were transferred to nitro cellulose membranes and blocked for 1 h at room temperature in Tris buffered saline with Tween 20 (TBST; 20mM Tris, 140 mM NaCl, 0.1 % Tween, pH 7.6) with 5 % non-fat milk powder and subsequently incubated with primary antibodies (1:1000–1:2000 dilutions). Bound antibodies were detected by horseradish peroxidase-coupled anti-rabbit IgG and visualized by using enhanced chemiluminescence (ECL; Picomax, Rockland) as described previously [92].

### RT-PCR analyses of aquaporin expression

Total RNA was extracted from RNA later stabilized *L. salmonis* from different developmental stages; fertilized eggs, nauplia 1 and 2, copepods, chalimus 1 and 2, preadult 1 and 2 males and females, and adult males and females with the RNAeasy Mini Kit (Qiagen). The RNA was DNAse treated before 80 ng was used for cDNA synthesis (as described above). 2 ul of 20-fold dilluted cDNA was used for template in 25 μL RT-PCR reactions using Gotaq DNA polymerase (Promega) and primer concentrations of 0.4 μM. (Primers are listed in Additional file 1: Table S3). 5uL of each PCR reaction (35 cycles) was run on 1 % agarose gels stained with Gelred^TM^ (Biotum) in TAE-buffer, and PCR expression products were visualised with UV-light in a gel doc imaging system (Biorad). To verify specific amplification the PCR products were sequenced following the BigDye protocol (v3.1).

### Availability of supporting data

All the supporting data are included as additional files.

## Additional file

Additional file 1: Figure S1. Extracellular views of cartoon renders of salmon louse aquaporins illustrating the ar/R constriction residues (spacefill) that determine the molecular selectivity of the channel. Labels are annotated as for Fig. 1 in the main text. **Figure S2.** Bayesian majority-rule mid-point rooted tree of an amino acid alignment of expressed transcripts in Maxillipoda. The tree was inferred from 500,000 MCMC generations on 8,085 amino acid sites. Posterior probabilities are shown at each node. Scale bar represents the number of amino acid substitutions per site. Grey bars highlight species with N-terminal splice-variants. **Figure S3.** Bayesian majority-rule mid-point rooted tree of a codon alignment of the aquaporin superfamilies in the Atlantic salmon and salmon louse. The tree was inferred from 1 million MCMC generations on 57,075 nucleotide sites. Posterior probabilities of the codon/amino acid analyses are shown at each node. Scale bar represents the number of nucleotide substitutions per site. **Figure S4.** In the absence of cAMP, salmon louse Glp1_v1 is non functional in *X. laevis* oocytes. (A) Osmotic water permeability (*P*_f_) of Glp1_v1 without the addition of cAMP compared to water- and *glp_3v2*-injected oocytes as negative and positve controls, respectively. (B) Immunofluorescence micrographs of paraffin sections of water- (Ctrl) and *glp1_v1*-injected oocytes in the absence of cAMP probed with paralog-specific antisera followed by Cy3-labeled anti-rabbit IgG. Arrows point to the plasma membrane. Inset: Magnified region shows the retention of Glp1_v1 (green) just below the oocyte plasma membrane. **Table S1.** C-terminal peptides used to immunize rabbits to raise salmon louse affinity-purified antibodies. **Table S2.** List of aquaporin accession numbers used in the study. **Table S3.** Oligonucleotide primers used for RT-PCR analysis. Nucleic acid sequences for primers specific for each salmon louse aquaporin mRNA, *ef1α* and expected product size. (PDF 2063 kb)

## Abbreviations

ANOVA: Analysis of variance
Aqp: Aquaporin
ar/R: Aromatic-arginine constriction
Bib: Big brain
BLAST: Basic local alignment search tool
cAMP: Cyclic adenosine monophosphate
Drip: Drosophila intrinsic protein
Eglp: Entomoglyceroporin
ELISA: Enzyme linked immunosorbant assay
Glp: Aquaglyceroporin
MAFFT: Multiple alignment using fast fourier transform
MBS: Barth’s culture medium
MCMC: Markov chain Monte Carlo
NPA: Asparagine-Proline-Alanine motif
ORF: Open reading frame
PBS: Phosphate-buffered saline
PCR: Ploymerase chain reaction
PripL: *Pyrocoelia rufa* integral protein-like
RACE: Rapid amplification of cDNA ends
RT: Reverse transcriptase
TBST: Tris buffered saline with Tween 20
t-coffee: Tree-based consistency objective function for alignment evaluation
TMD: Transmembrane domain
UTR: Untranslated region.
