# Supplementary material for: Phylogenomic and functional analyses of salmon lice aquaporins uncover the molecular diversity of the superfamily in Arthropoda
Source: BMC Genomics. 2015 Aug 19;16(1):618. doi: 10.1186/s12864-015-1814-8 (PMC4539701; doi:10.1186/s12864-015-1814-8)
Supplement: Additional file 1: Figure S1. — Extracellular views of cartoon renders of salmon louse aquaporins illustrating the ar/R constriction residues (spacefill) that determine the molecular selectivity of the channel. Labels are annotated as for Fig. 1 in the main text. Figure S2. Bayesian majority-rule mid-point rooted tree of an amino acid alignment of expressed transcripts in Maxillipoda. The tree was inferred from 500,000 MCMC generations on 8,085 amino acid sites. Posterior probabilities are shown at each node. Scale bar represents the number of amino acid substitutions per site. Grey bars highlight species with N-terminal splice-variants. Figure S3. Bayesian majority-rule mid-point rooted tree of a codon alignment of the aquaporin superfamilies in the Atlantic salmon and salmon louse. The tree was inferred from 1 million MCMC generations on 57,075 nucleotide sites. Posterior probabilities of the codon/amino acid analyses are shown at each node. Scale bar represents the number of nucleotide substitutions per site. Figure S4. In the absence of cAMP, salmon louse Glp1_v1 is non functional in X. laevis oocytes. (A) Osmotic water permeability (P f) of Glp1_v1 without the addition of cAMP compared to water- and glp_3v2-injected oocytes as negative and positve controls, respectively. (B) Immunofluorescence micrographs of paraffin sections of water- (Ctrl) and glp1_v1-injected oocytes in the absence of cAMP probed with paralog-specific antisera followed by Cy3-labeled anti-rabbit IgG. Arrows point to the plasma membrane. Inset: Magnified region shows the retention of Glp1_v1 (green) just below the oocyte plasma membrane. Table S1. C-terminal peptides used to immunize rabbits to raise salmon louse affinity-purified antibodies. Table S2. List of aquaporin accession numbers used in the study. Table S3. Oligonucleotide primers used for RT-PCR analysis. Nucleic acid sequences for primers specific for each salmon louse aquaporin mRNA, ef1α and expected product size. (PDF 2063 kb) [file 12864_2015_1814_MOESM1_ESM.pdf]

Supplementary data for

Phylogenomic and functional analyses of salmon lice aquaporins uncover the molecular diversity of the superfamily in Arthropoda

by

Jon Anders Stavang [1] (jon.stavang@uib.no), Francois Chauvigné [1,3] [*](francois.chauvigne@uib.no), Heidi Kongshaug [1](heidi.kongshaug@uib.no), Joan Cerdà [3] (joan.cerda@irta.cat), Frank Nilsen [1] (frank.nilsen@uib.no), Roderick Nigel Finn [1,2 *] (nigel.finn@uib.no)

Supplemental information

Additional file 1

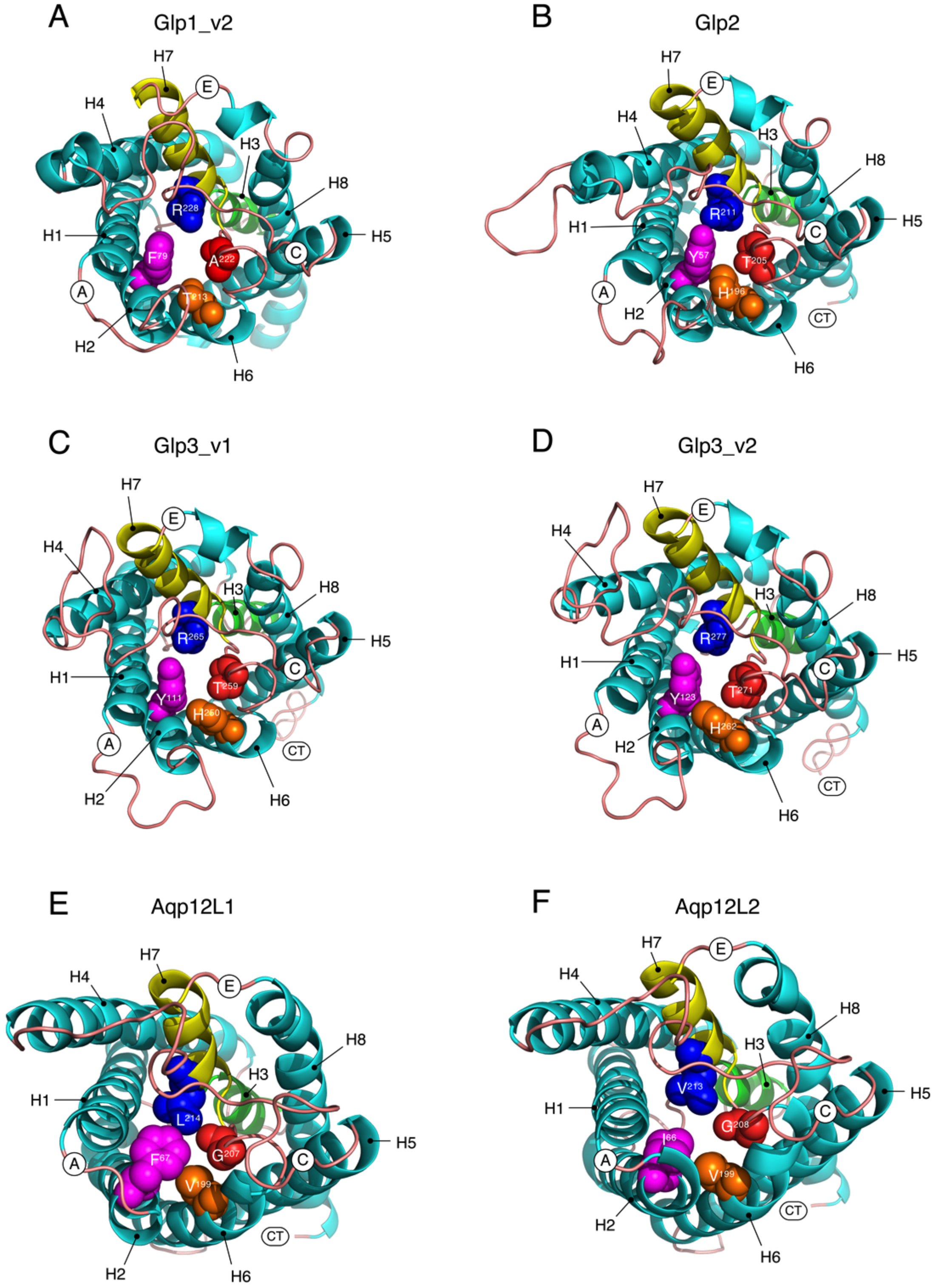

Figure S1. Extracellular views of cartoon renders of salmon louse aquaporins illustrating the ar/R constriction residues (spacefill) that determine the molecular selectivity of the channel. Labels are annotated as for Figure 1 in the main text.

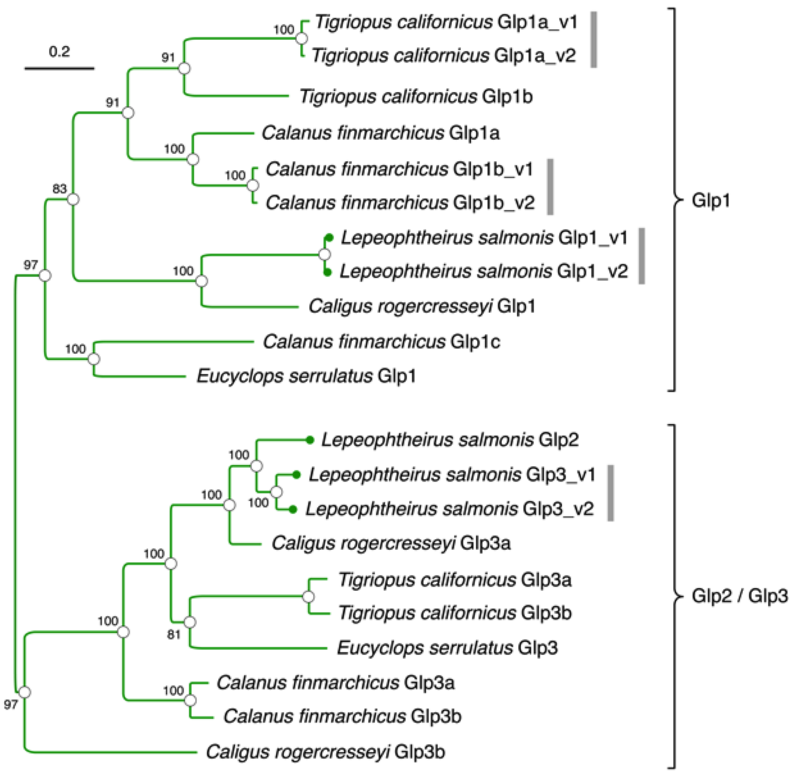

Figure S2. Bayesian majority-rule mid-point rooted tree of an amino acid alignment of expressed transcripts in Maxillipoda. The tree was inferred from 500,000 MCMC generations on 8,085 amino acid sites. Posterior probabilities are shown at each node. Scale bar represents the number of amino acid substitutions per site. Grey bars highlight species with N-terminal splice-variants

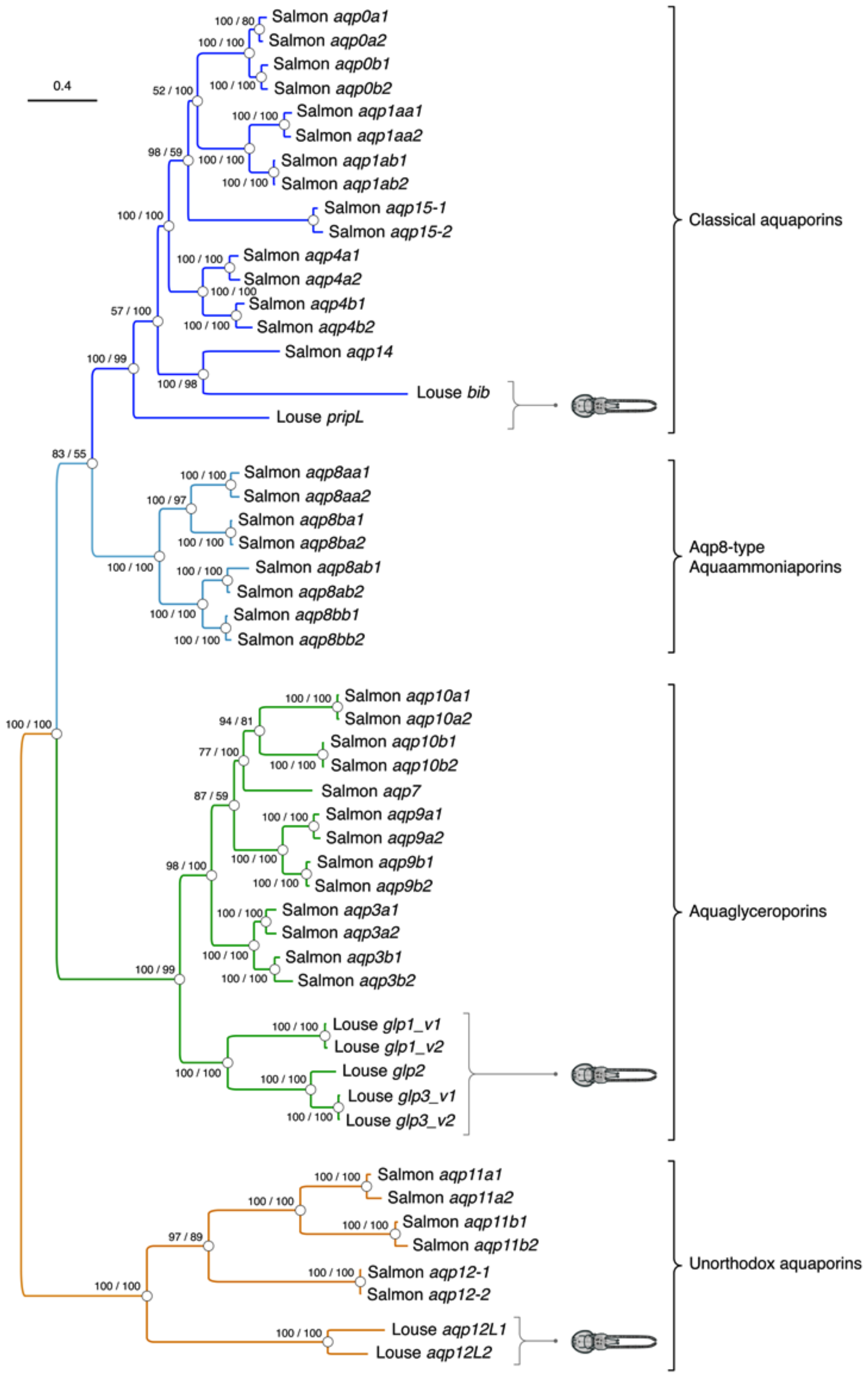

Figure S3. Bayesian majority-rule mid-point rooted tree of a codon alignment of the aquaporin superfamilies in the Atlantic salmon and salmon louse. The tree was inferred from 1 million MCMC generations on 57,075 nucleotide sites. Posterior probabilities of the codon/amino acid analyses are shown at each node. Scale bar represents the number of nucleotide substitutions per site.

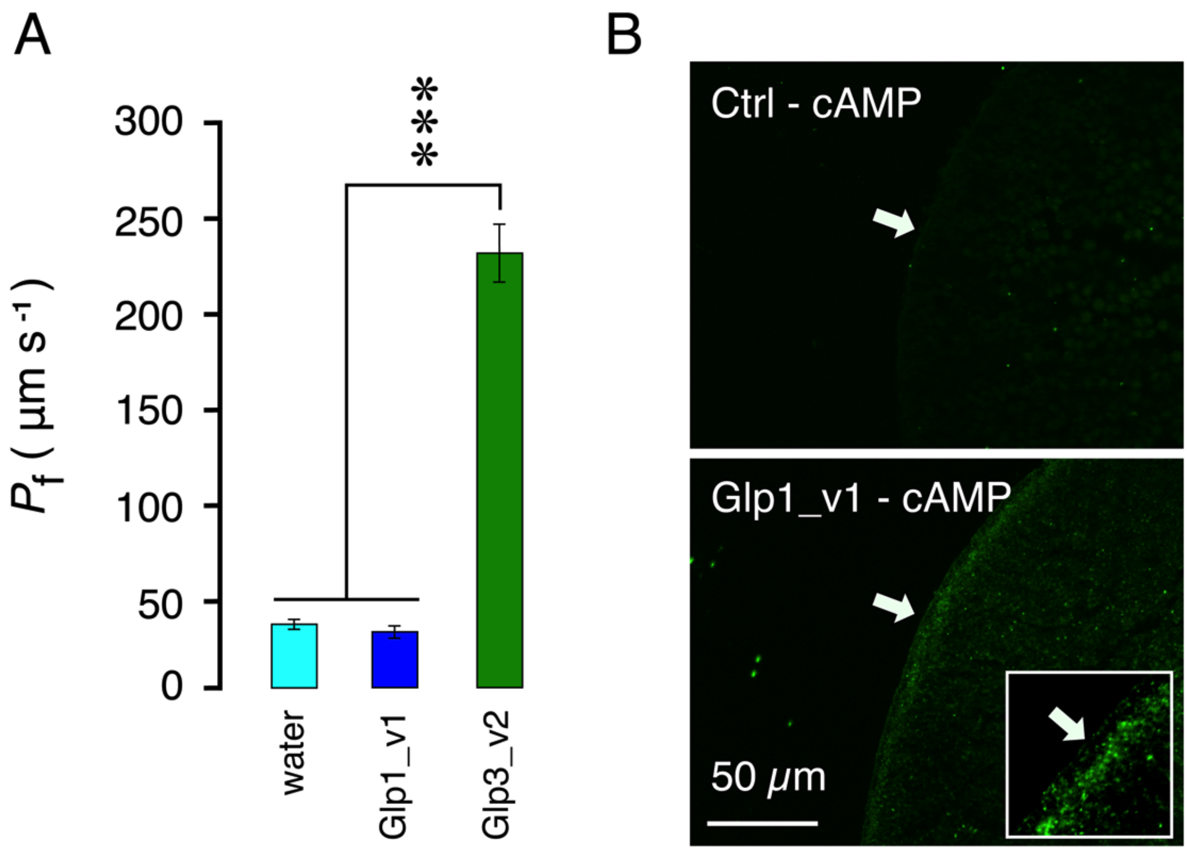

Figure S4. In the absence of cAMP, salmon louse Glp1_v1 is non functional in *X. laevis* oocytes. (A) Osmotic water permeability (P_f) of Glp1_v1 without the addition of cAMP compared to water- and *glp_3v2*-injected oocytes as negative and positve controls, respectively. (B) Immunofluorescence micrographs of paraffin sections of water- (Ctrl) and *glp1_v1*-injected oocytes in the absence of cAMP probed with paralog-specific antisera followed by Cy3-labeled anti-rabbit IgG. Arrows point to the plasma membrane. Inset: Magnified region shows the retention of Glp1_v1 (green) just below the oocyte plasma membrane.

Table S1: C-terminal peptides used to immunize rabbits to raise salmon louse affinity-purified antibodies

Paralog	Antigen
Bib-Ct	(NH_2-) CSRHRGDFYSPSYNA (-$CONH_2$)
PripL-Ct	(NH_2-) CRPSHSLAEEYGIVRT (-COOH)
Glp1-Ct	(NH_2-) CSLEDQRTPNLIMNL (-COOH)
Glp3-Ct	(NH_2-) C(Ahx)IELHHPIDE (-COOH)
Aqp12L1-Ct	(NH_2-) CVFNYTTKAKEE (-COOH)
Aqp12L2-Ct	(NH_2-) CLSDFNSKKSKVN (-COOH)

Table S2. List of aquaporin accession numbers used in the study (see following pages)

Table S3: Oligonucleotide primers used for RT-PCR analysis. Nucleic acid sequences for primers specific for each salmon louse aquaporin mRNA, *ef1α* and expected product size.

Gene	Forward	Reverse	Expected Product size
bib	CGCAATCCTCCTTATCCAGA	ATTTGCTCCCAAGTTCTCCA	112
pripL	CCATTGGCCATTGGATTAGC	AATAACAGCCGGTCCAAATG	102
glp1_v1	AAGCGAAAGAAGAGTAACTTAGTG	ACCCATATGATTGTCAAACAGC	112
glp1_v2	TCCCTCGTCATTCTAGCTTTC	GTATATGGTCCGTCAATGGATG	106
glp2	AACCTTATCACTCACGACTAAC	CAAGGATGGTCATTCTCGAATTG	115
glp3_v1	AGATTGAAGTGGGAAACATGTC	CAAGGATGGTCATTCTCGAATTG	100
glp3_v2	ACTTTGCTCCACGATTATTCAC	CAAGAACTCCACCCACATGA	114
aqp12L1	GAGAAACCAGTGAAAGGATTATTG	GGTCATCATGATGCCTTCTG	136
aqp12L2	ACATTCAGAAGGAGAAGGACAA	ACTTTAGGAACACTGGTCATCAT	117
ef1α	CATCGCCTGCAAGTTTAACCAAATT	CCGGCATCACCAGACTTGA	117

Additional file: Table S2: Accession numbers of sequences analysed in the study

Accession #	ortholog	Animal	Species	Rank	Order	Family
**Atlantic salmon paralogs**						
KM677197	AQP 0a1	Atlantic salmon	*Salmo salar*	Protacanthopterygii	Salmoniformes	Salmonidae
KM677198	AQP 0a2	Atlantic salmon	*Salmo salar*	Protacanthopterygii	Salmoniformes	Salmonidae
KM677199	AQP 0b1	Atlantic salmon	*Salmo salar*	Protacanthopterygii	Salmoniformes	Salmonidae
KM677200	AQP 0b2	Atlantic salmon	*Salmo salar*	Protacanthopterygii	Salmoniformes	Salmonidae
AGKD01005533/ACI66426	AQP 1aa1	Atlantic salmon	*Salmo salar*	Protacanthopterygii	Salmoniformes	Salmonidae
AGKD01009244/ACI67627	AQP 1aa2	Atlantic salmon	*Salmo salar*	Protacanthopterygii	Salmoniformes	Salmonidae
AGKD01338342	AQP 1ab1	Atlantic salmon	*Salmo salar*	Protacanthopterygii	Salmoniformes	Salmonidae
ACI33306/NP_001133472/AGKD01009244	AQP 1ab2	Atlantic salmon	*Salmo salar*	Protacanthopterygii	Salmoniformes	Salmonidae
AGKD01027288	AQP 15-1	Atlantic salmon	*Salmo salar*	Protacanthopterygii	Salmoniformes	Salmonidae
AGKD01030871/AGKD01079866	AQP 15-2	Atlantic salmon	*Salmo salar*	Protacanthopterygii	Salmoniformes	Salmonidae
AGKD01106121	AQP 4a1	Atlantic salmon	*Salmo salar*	Protacanthopterygii	Salmoniformes	Salmonidae
AGKD01009294	AQP 4a2	Atlantic salmon	*Salmo salar*	Protacanthopterygii	Salmoniformes	Salmonidae
AGKD01074371	AQP 4b1	Atlantic salmon	*Salmo salar*	Protacanthopterygii	Salmoniformes	Salmonidae
AGKD01045191	AQP 4b2	Atlantic salmon	*Salmo salar*	Protacanthopterygii	Salmoniformes	Salmonidae
AGKD01272622/AGKD01346587	AQP 14-2	Atlantic salmon	*Salmo salar*	Protacanthopterygii	Salmoniformes	Salmonidae
AGKD01005999/KC626878	AQP 8aa1	Atlantic salmon	*Salmo salar*	Protacanthopterygii	Salmoniformes	Salmonidae
AGKD01093852/AGKD01051269/DW573347	AQP 8aa2	Atlantic salmon	*Salmo salar*	Protacanthopterygii	Salmoniformes	Salmonidae
AGKD01005999/DW532465	AQP 8ab1	Atlantic salmon	*Salmo salar*	Protacanthopterygii	Salmoniformes	Salmonidae
AGKD01051269/KC626879	AQP 8ab2	Atlantic salmon	*Salmo salar*	Protacanthopterygii	Salmoniformes	Salmonidae
AGKD01042454	AQP 8ba1	Atlantic salmon	*Salmo salar*	Protacanthopterygii	Salmoniformes	Salmonidae
AGKD01119048/AGKD01190352	AQP 8ba2	Atlantic salmon	*Salmo salar*	Protacanthopterygii	Salmoniformes	Salmonidae
AGKD01119048/AGKD01156665/KC626880/ACN11279	AQP 8bb1	Atlantic salmon	*Salmo salar*	Protacanthopterygii	Salmoniformes	Salmonidae
AGKD01042454	AQP 8bb2	Atlantic salmon	*Salmo salar*	Protacanthopterygii	Salmoniformes	Salmonidae
AGKD01094322/AGKD01056897	AQP 3a1	Atlantic salmon	*Salmo salar*	Protacanthopterygii	Salmoniformes	Salmonidae
AGKD01068849/AGKD01149541	AQP 3a2	Atlantic salmon	*Salmo salar*	Protacanthopterygii	Salmoniformes	Salmonidae
AGKD01002624	AQP 3b1	Atlantic salmon	*Salmo salar*	Protacanthopterygii	Salmoniformes	Salmonidae
AGKD01215587	AQP 3b2	Atlantic salmon	*Salmo salar*	Protacanthopterygii	Salmoniformes	Salmonidae
AGKD01252585/AGKD01126390/AGKD01010521	AQP 9a1	Atlantic salmon	*Salmo salar*	Protacanthopterygii	Salmoniformes	Salmonidae
AGKD01109773/AGKD01265939/AGKD01211321/AGKD01370165 /AGKD01142451	AQP 9a2	Atlantic salmon	*Salmo salar*	Protacanthopterygii	Salmoniformes	Salmonidae
AGKD01025380/AGKD01043301	AQP 9b1	Atlantic salmon	*Salmo salar*	Protacanthopterygii	Salmoniformes	Salmonidae
AGKD01037951/AGKD01133133/AGKD01006400	AQP 9b2	Atlantic salmon	*Salmo salar*	Protacanthopterygii	Salmoniformes	Salmonidae
AGKD01021376/AGKD01033103/AGKD01065234	AQP 7	Atlantic salmon	*Salmo salar*	Protacanthopterygii	Salmoniformes	Salmonidae
AGKD01044249	AQP 10a1	Atlantic salmon	*Salmo salar*	Protacanthopterygii	Salmoniformes	Salmonidae
AGKD01200314	AQP 10a2	Atlantic salmon	*Salmo salar*	Protacanthopterygii	Salmoniformes	Salmonidae
AGKD01090696	AQP 10b1	Atlantic salmon	*Salmo salar*	Protacanthopterygii	Salmoniformes	Salmonidae
AGKD01157343	AQP 10b2	Atlantic salmon	*Salmo salar*	Protacanthopterygii	Salmoniformes	Salmonidae
EZ807701/EZ814810/CCAF010005608	AQP 11a1	Atlantic salmon	*Salmo salar*	Protacanthopterygii	Salmoniformes	Salmonidae
AGKD01029804	AQP 11a2	Atlantic salmon	*Salmo salar*	Protacanthopterygii	Salmoniformes	Salmonidae
AGKD01193048	AQP 11b1	Atlantic salmon	*Salmo salar*	Protacanthopterygii	Salmoniformes	Salmonidae
AGKD01088157	AQP 11b2	Atlantic salmon	*Salmo salar*	Protacanthopterygii	Salmoniformes	Salmonidae
AGKD01402623	AQP 12-1	Atlantic salmon	*Salmo salar*	Protacanthopterygii	Salmoniformes	Salmonidae
AGKD01127630	Aqp 12-2	Atlantic salmon	*Salmo salar*	Protacanthopterygii	Salmoniformes	Salmonidae
**Arthropoda Bib**						
FBpp0079519	Bib	Fruit fly	*Drosophila melanogaster*	Hexapoda	Diptera	Drosophilidae
FBpp0222049	Bib	Fruit fly	*Drosophila simulans*	Hexapoda	Diptera	Drosophilidae
FBpp0199328	Bib	Fruit fly	*Drosophila sechellia*	Hexapoda	Diptera	Drosophilidae
FBpp0263898	Bib	Fruit fly	*Drosophila yakuba*	Hexapoda	Diptera	Drosophilidae
FBpp0128621	Bib	Fruit fly	*Drosophila erecta*	Hexapoda	Diptera	Drosophilidae
FBpp0118926	Bib	Fruit fly	*Drosophila ananassae*	Hexapoda	Diptera	Drosophilidae
FBpp0280277	Bib	Fruit fly	*Drosophila pseudoobscura*	Hexapoda	Diptera	Drosophilidae
FBpp0183059	Bib	Fruit fly	*Drosophila persimilis*	Hexapoda	Diptera	Drosophilidae
ABL85281	Bib	Fruit fly	*Drosophila americana*	Hexapoda	Diptera	Drosophilidae
FBpp0232341	Bib	Fruit fly	*Drosophila virilis*	Hexapoda	Diptera	Drosophilidae
FBpp0166799	Bib	Fruit fly	*Drosophila mojavensis*	Hexapoda	Diptera	Drosophilidae
FBpp0144970	Bib	Fruit fly	*Drosophila grimshawi*	Hexapoda	Diptera	Drosophilidae
FBpp0244725/FBpp0241572	Bib	Fruit fly	*Drosophila willistoni*	Hexapoda	Diptera	Drosophilidae
AFP49901	Bib	Tsetse fly	*Glossina morsitans morsitans*	Hexapoda	Diptera	Glossinidae
XP_004534536	Bib	Mediterranean fruit fly	*Ceratitis capitata*	Hexapoda	Diptera	Tephritidae
AAEL004741/XP_001649747	Bib	Yellow fever mosquito	*Aedes aegypti*	Hexapoda	Diptera	Culicidae
CPIJ016447	Bib	Southern house mosquito	*Culex quinquefasciatus*	Hexapoda	Diptera	Culicidae
AGAP008766/AGAP008767	Bib	African malaria mosquito	*Anopheles gambiae*	Hexapoda	Diptera	Culicidae
EFR20654/ETN57779	Bib	American malaria mosquito	*Anopheles darlingi*	Hexapoda	Diptera	Culicidae
GAAK01005117/GAAK01001577	Bib	Antarctic flightless midge	*Belgica antarctica*	Hexapoda	Diptera	Chironomidae
KA181207	Bib	Harlequin fly	*Chironomus riparius*	Hexapoda	Diptera	Chironomidae
AADK01007500/BABH01027418/BABH01043412	Bib	Domestic silkworm	*Bombyx mori*	Hexapoda	Lepidoptera	Bombycidae
CAEZ01006773	Bib	Postman butterfly	*Heliconius melpomene melpomene*	Hexapoda	Lepidoptera	Nymphalidae
EHJ64738/EHJ63154	Bib	Monarch butterfly	*Danaus plexippus*	Hexapoda	Lepidoptera	Nymphalidae
AIXA01000727/AIXA01000726/AIXA01003168	Bib	Tobacco hornworm	*Manduca sexta*	Hexapoda	Lepidoptera	Sphingidae
TC010832	Bib	Red flour Beetle	*Tribolium castaneum*	Hexapoda	Coleoptera	Tenebrionidae
JU421678	Bib	Salt marsh beetle	*Pogonus chalceus*	Hexapoda	Coleoptera	Carabidae
JR479665	Bib	Red palm weevil	*Rhynchophorus ferrugineus*	Hexapoda	Coleoptera	Curculionidae
ENN74136	Bib	Mountain pine beetle	*Dendroctonus ponderosae*	Hexapoda	Coleoptera	Curculionidae
XP_003399074	Bib	Buff-tailed bumblebee	*Bombus terrestris*	Hexapoda	Hymenoptera	Apidae
XP_003487862	Bib	Common eastern bumble bee	*Bombus impatiens*	Hexapoda	Hymenoptera	Apidae
XP_396705	Bib	Honey bee	*Apis mellifera*	Hexapoda	Hymenoptera	Apidae
XP_003699287	Bib	Alfalfa leafcutting bee	*Megachile rotundata*	Hexapoda	Hymenoptera	Megachilidae
EFZ15059	Bib	Red fire ant	*Solenopsis invicta*	Hexapoda	Hymenoptera	Formicidae
AEAB01004397	Bib	Florida carpenter ant	*Camponotus floridanus*	Hexapoda	Hymenoptera	Formicidae
EFN86731/EFN86732	Bib	Jerdon's jumping ant	*Harpegnathos saltator*	Hexapoda	Hymenoptera	Formicidae
EZA50845	Bib	Clonal raider ant	*Cerapachys biroi*	Hexapoda	Hymenoptera	Formicidae
EGI69716/AEVX01004595	Bib	Panamanian leafcutter ant	*Acromyrmex echinatior*	Hexapoda	Hymenoptera	Formicidae
ACEP_00006876	Bib	Leafcutter ant	*Atta cephalotes*	Hexapoda	Hymenoptera	Formicidae
XP_001604170	Bib	Jewel wasp	*Nasonia vitripennis*	Hexapoda	Hymenoptera	Pteromalidae
ADAO01027634/ADAO01027617	Bib	Jewel wasp	*Nasonia giraulti*	Hexapoda	Hymenoptera	Pteromalidae
XP_002433221	Bib	Human body louse	*Pediculus humanus corporis*	Hexapoda	Phthiraptera	Pediculidae
ACPB02031565/ACPB02009321	Bib	Assassin bug	*Rhodnius prolixus*	Hexapoda	Hemiptera	Reduviidae
XP_001948407	Bib	Pea aphid	*Acyrthosiphon pisum*	Hexapoda	Hemiptera	Aphididae
GACJ01023397	Bib	Asian citrus psyllid	*Diaphorina citri*	Hexapoda	Hemiptera	Psyllidae
JPZV01159195/JPZV01159181/JPZV01159174	Bib	German cockroach	*Blattella germanica*	Hexapoda	Blattodea	Ectobiidae
GAWS01120948	Bib	American cockroach	*Periplaneta americana*	Hexapoda	Blattodea	Blattidae
GAYD01311266	Bib	Blaberus cockroach	*Blaberus atropos*	Hexapoda	Blattodea	Blaberidae
GAZN01184473	Bib	Brown hooded cockroach	*Cryptocercus wrighti*	Hexapoda	Blattodea	Cryptocercidae
GASE01250065	Bib	Cuban subterranean termite	*Prorhinotermes simplex*	Hexapoda	Isoptera	Rhinotermitidae

AUST01019796/AUST01019797	Bib	Nevada dampwood termite	*Zootermopsis nevadensis*	Hexapoda	Isoptera	Termopsidae
GASW01145900	Bib	Praying mantis	*Mantis religiosa*	Hexapoda	Mantodea	Mantidae
GAYA01368224	Bib	Zoraptid	*Zorotypus caudelli*	Hexapoda	Orthoptera	Zorotypidae
GAWU01257959	Bib	Webspinner	*Aposthonia japonica*	Hexapoda	Embioptera	Oligotomidae
GAWG01145562	Bib	Giant prickly stick insect	*Extatosoma tiaratum*	Hexapoda	Phasmatodea	Phasmatidae
GAWC01014738	Bib	Thorny stick insect	*Aretaon asperrimus*	Hexapoda	Phasmatodea	Heteropterygidae
GAUX01120890	Bib	Camel cricket	*Ceuthophilus sp.*	Hexapoda	Orthoptera	Rhaphidophoridae
GAZT01171842	Bib	False stick insect	*Prosarthria teretrirostris*	Hexapoda	Orthoptera	Proscopiidae
GBHB01007420	Bib	Oceanic field cricket	*Teleogryllus commodus*	Hexapoda	Orthoptera	Gryllidae
GASQ01013559	Bib	Slender Groundhopper	*Tetrix subulata*	Hexapoda	Orthoptera	Tetrigidae
GAUZ01380599	Bib	Stripe-winged grasshopper	*Stenobothrus lineatus*	Hexapoda	Orthoptera	Acrididae
AVCP010869913	Bib	Migratory locust	*Locusta migratoria*	Hexapoda	Orthoptera	Acrididae
GATU01061203	Bib	Blue-winged olive	*Baetis sp.*	Hexapoda	Ephemeroptera	Baetidae
GAXA01112850	Bib	Mahogany Dun	*Isonychia bicolor*	Hexapoda	Ephemeroptera	Isonychiidae
GAZG01101625	Bib	Mayfly	*Eurylophella sp.*	Hexapoda	Ephemeroptera	Ephemerellidae
AYNC01046590/AYNC01046589	Bib	Green drake	*Ephemera danica*	Hexapoda	Ephemeroptera	Ephemeridae
GAYO01122019	Bib	Golden-ringed dragonfly	*Cordulegaster boltonii*	Hexapoda	Odonata	Cordulegastridae
APVN01095476/APVN01095473	Bib	Scarce chaser	*Ladona fulva*	Hexapoda	Odonata	Libellulidae
GASN01409838	Bib	Firebrat	*Thermobia domestica*	Hexapoda	Zygentoma	Lepismatidae
GAYJ01257237	Bib	Silverfish	*Atelura formicaria*	Hexapoda	Zygentoma	Nicoletiidae
GASO01228638	Bib	Silverfish	*Tricholepidion gertschi*	Hexapoda	Zygentoma	Libellulidae
GAUM01007766	Bib	Bristletail	*Machilis hrabei*	Hexapoda	Archaeognatha	Machilidae
GAUG01018425	Bib	Bristletail	*Meinertellus cundinamarcensis*	Hexapoda	Archaeognatha	Meinertellidae
GAXJ01108916	Bib	Two-pronged bristletail	*Occasjapyx japonicus*	Hexapoda	Diplura	Japygidae
GAXE01131619	Bib	Conehead	*Acerentomon sp.*	Hexapoda	Protura	Acerentomidae
AFFK01019694/AFFK01019695/SMAR015755	Bib	Coastal European centipede	*Strigamia maritima*	Chilopoda	Geophilomorpha	Linotaeniidae
GAFS01004822/GAFY01027786	Bib	Narrow-clawed crayfish	*Pontastacus leptodactylus*	Malacostraca	Decapoda	Astacidae
GADE01005115	Bib	Australian red claw crayfish	*Cherax quadricarinatus*	Malacostraca	Decapoda	Parastacidae
FE778057	Bib	Flat porcelain crab	*Petrolisthes cinctipes*	Malacostraca	Decapoda	Porcellanidae
DV467482	Bib	Green shore Crab	*Carcinus maenas*	Malacostraca	Decapoda	Carcinidae
JW508536	Bib	Tide pool copepod	*Tigriopus californicus*	Maxillopoda	Harpacticoida	Harpacticidae
KR005658	Bib	Salmon louse	*Lepeophtheirus salmonis*	Maxillopoda	Siphonostomatoida	Caligidae
JW965321	Bib	Argulus	*Argulus siamensis*	Maxillopoda	Arguloida	Argulidae
EFX82861	Bib	Common water flea	*Daphnia pulex*	Branchiopoda	Diplostraca	Daphniidae
XP_002408745/XP_002408744	Bib	Black-legged tick	*Ixodes scapularis*	Acari	Ixodida	Ixodidae
GACK01002509	Bib	Zebra tick	*Rhipicephalus pulchellus*	Acari	Ixodida	Ixodidae
CK185295	Bib	Southern cattle tick	*Rhipicephalus microplus*	Acari	Ixodida	Ixodidae
GW003922	Bib	Two-spotted spider mite	*Tetranychus urticae*	Acari	Acariformes	Tetranychidae
AZAQ01093526/AZAQ01093525/AZAQ01093524/GAZR01022102	Bib	African social eresid spider	*Stegodyphus mimosarum*	Arachnida	Araneae	Eresidae
GBCS01010810	Bib	Western black widow	*Latrodectus hesperus*	Arachnida	Araneae	Theridiidae
AOMJ01042817/AOMJ01089836/AOMJ01042847/AOMJ01042849	Bib	Common house spider	*Parasteatoda tepidariorum*	Arachnida	Araneae	Theridiidae
AYEL01087177	Bib	Chinese scorpion	*Mesobuthus martensii*	Arachnida	Scorpiones	Buthidae
AXZI01186657	Bib	Baja California bark scorpion	*Centruroides exilicauda*	Arachnida	Scorpiones	Buthidae

Hexapoda Drip

FBpp0087240	Drip	Fruit fly	*Drosophila melanogaster*	Hexapoda	Diptera	Drosophilidae
FBpp0201903	Drip	Fruit fly	*Drosophila sechellia*	Hexapoda	Diptera	Drosophilidae
FBpp0258530	Drip	Fruit fly	*Drosophila yakuba*	Hexapoda	Diptera	Drosophilidae
FBpp0141192	Drip	Fruit fly	*Drosophila erecta*	Hexapoda	Diptera	Drosophilidae
FBpp0115586	Drip	Fruit fly	*Drosophila ananassae*	Hexapoda	Diptera	Drosophilidae
FBpp0276838	Drip	Fruit fly	*Drosophila pseudoobscura*	Hexapoda	Diptera	Drosophilidae
FBpp0236388	Drip	Fruit fly	*Drosophila virilis*	Hexapoda	Diptera	Drosophilidae
FBpp0170266	Drip	Fruit fly	*Drosophila mojavensis*	Hexapoda	Diptera	Drosophilidae
FBpp0154409	Drip	Fruit fly	*Drosophila grimshawi*	Hexapoda	Diptera	Drosophilidae
FBpp0251027	Drip	Fruit fly	*Drosophila willistoni*	Hexapoda	Diptera	Drosophilidae
BAM26200	Drip	Black blowfly	*Phormia regina*	Hexapoda	Diptera	Calliphoridae
AEG47703	Drip	Oriental latrine fly	*Chrysomya megacephala*	Hexapoda	Diptera	Calliphoridae
Q25074	Drip	Buffalo fly	*Haematobia irritans exigua*	Hexapoda	Diptera	Muscidae
ADD19102	Drip 1	Tsetse fly	*Glossina morsitans morsitans*	Hexapoda	Diptera	Glossinidae
ADD20051	Drip 2	Tsetse fly	*Glossina morsitans morsitans*	Hexapoda	Diptera	Glossinidae
FG293007/FG295048	Drip	Primary screw-worm	*Cochliomyia hominivorax*	Hexapoda	Diptera	Calliphoridae
AAEL003512/XP_001656931	Drip	Yellow fever mosquito	*Aedes aegypti*	Hexapoda	Diptera	Culicidae
CPIJ015704/CPIJ015704	Drip	Southern house mosquito	*Culex quinquefasciatus*	Hexapoda	Diptera	Culicidae
JAA93938	Drip	Psorophora mosquito	*Psorophora albipes*	Hexapoda	Diptera	Culicidae
AGAP008842	Drip	African malaria mosquito	*Anopheles gambiae*	Hexapoda	Diptera	Culicidae
EZ976133	Drip	African malaria mosquito	*Anopheles funestus*	Hexapoda	Diptera	Culicidae
GAAK01006851/GAAK01006849	Drip	Antarctic flightless midge	*Belgica antarctica*	Hexapoda	Diptera	Chironomidae
ABV60346	Drip	Sand fly	*Lutzomyia longipalpis*	Hexapoda	Diptera	Psychodidae
GAKJ01008613	Drip	Orange wheat blossom midge	*Sitodiplosis mosellana*	Hexapoda	Diptera	Cecidomyiidae
AB178640	Drip	Domestic silkworm	*Bombyx mori*	Hexapoda	Lepidoptera	Bombycidae
CAEZ01007264/CAEZ01007265	Drip	Postman butterfly	*Heliconius melpomene melpomene*	Hexapoda	Lepidoptera	Nymphalidae
EHJ75085	Drip	Monarch butterfly	*Danaus plexippus*	Hexapoda	Lepidoptera	Nymphalidae
FQ019249	Drip	African cotton leafworm	*Spodoptera littoralis*	Hexapoda	Lepidoptera	Noctuidae
GAFU01003217	Drip	Beet armyworm	*Spodoptera exigua*	Hexapoda	Lepidoptera	Noctuidae
HO053923	Drip	Tobacco budworm	*Heliothis virescens*	Hexapoda	Lepidoptera	Noctuidae
EZ583816	Drip	western bean cutworm	*Striacosta albicosta*	Hexapoda	Lepidoptera	Noctuidae
BAM19007	Drip	Common mormon	*Papilio polytes*	Hexapoda	Lepidoptera	Papilionidae
BAM17858	Drip	Asian Swallowtail	*Papilio xuthus*	Hexapoda	Lepidoptera	Papilionidae
AFC34081	Drip	striped riceborer	*Chilo suppressalis*	Hexapoda	Lepidoptera	Crambidae
JP612973	Drip	Propertius duskywing	*Erynnis propertius*	Hexapoda	Lepidoptera	Hesperiidae
JO817751/GR920919	Drip	Tobacco hornworm	*Manduca sexta*	Hexapoda	Lepidoptera	Sphingidae
BAH47554	Drip	Oriental fruit moth	*Grapholita molesta*	Hexapoda	Lepidoptera	Tortricidae
TC011257	Drip	Red flour Beetle	*Tribolium castaneum*	Hexapoda	Coleoptera	Tenebrionidae
BAM83568	Drip	Beetle	*Anomala cuprea*	Hexapoda	Coleoptera	Scarabaeidae
JR487579	Drip	Red palm weevil	*Rhynchophorus ferrugineus*	Hexapoda	Coleoptera	Curculionidae
AEE63193	Drip	Mountain pine weevil	*Dendroctonus ponderosae*	Hexapoda	Coleoptera	Curculionidae
JU414336	Drip	Salt marsh beetle	*Pogonus chalceus*	Hexapoda	Coleoptera	Carabidae
GAPE01027420	Drip	Pollen beetle	*Brassicogethes aeneus*	Hexapoda	Coleoptera	Nitidulidae
GAAB01000751	Drip	Emerald ash borer	*Agrilus planipennis*	Hexapoda	Coleoptera	Buprestidae
GAXW01087449	Drip	Antlion	*Euroleon nostras*	Hexapoda	Neoptera	Myrmeleontidae
GAVV01176955	Drip	Green lacewing	*Pseudomallada prasinus*	Hexapoda	Neoptera	Chrysopidae
XP_003394164	Drip	Buff-tailed bumblebee	*Bombus terrestris*	Hexapoda	Hymenoptera	Apidae
XP_003487533	Drip	Common eastern bumble bee	*Bombus impatiens*	Hexapoda	Hymenoptera	Apidae
XP_624531	Drip	Honey bee	*Apis mellifera*	Hexapoda	Hymenoptera	Apidae
XP_003701708	Drip	Alfalfa leafcutting bee	*Megachile rotundata*	Hexapoda	Hymenoptera	Megachilidae
AEAQ01012360/EFZ22501	Drip	Red fire ant	*Solenopsis invicta*	Hexapoda	Hymenoptera	Formicidae
AEAB01004718/EFN72836	Drip	Florida carpenter ant	*Camponotus floridanus*	Hexapoda	Hymenoptera	Formicidae
JP783671/JP783671	Drip	Caribbean crazy ant	*Nylanderia pubens*	Hexapoda	Hymenoptera	Formicidae
ADOQ01012968	Drip	Argentine ant	*Linepithema humile*	Hexapoda	Hymenoptera	Formicidae

XP_001607940	Drip	Jewel wasp	*Nasonia vitripennis*	Hexapoda	Hymenoptera	Pteromalidae
GAKG01000818	Drip	Diamondback moth parasitoid	*Cotesia vestalis*	Hexapoda	Hymenoptera	Braconidae
EEB12655	Drip	Human body louse	*Pediculus humanus corporis*	Hexapoda	Phthiraptera	Pediculidae
GAWR01000696	Drip	Poultry shaft louse	*Menopon gallinae*	Hexapoda	Phthiraptera	Menoponidae
GAYV01109739	Drip	Booklice	*Liposcelis bostrychophila*	Hexapoda	Psocoptera	Liposcelidae
GAPT01006904	Drip	Booklice	*Ectopsocus briggsi*	Hexapoda	Psocoptera	Ectopsocidae
GAXD01024860	Drip	Western flower thrips	*Frankliniella occidentalis*	Hexapoda	Thysanoptera	Thripidae
JAA75980	Drip	Assassin bug	*Rhodnius prolixus*	Hexapoda	Hemiptera	Reduviidae
KF048092	Drip	Lygus bug	*Lygus hesperus*	Hexapoda	Hemiptera	Miridae
GAJX01005354	Drip	Pod sucking bug	*Clavigralla tomentosicollis*	Hexapoda	Hemiptera	Coreidae
GAJW01004051	Drip	Cowpea aphid	*Aphis craccivora*	Hexapoda	Hemiptera	Aphididae
ACL01373	Drip	Pea aphid	*Acyrthosiphon pisum*	Hexapoda	Hemiptera	Aphididae
GAAF01000345	Drip	Potato aphid	*Macrosiphum euphorbiae*	Hexapoda	Hemiptera	Aphididae
EE571220	Drip	Green peach aphid	*Myzus persicae*	Hexapoda	Hemiptera	Aphididae
FO035865	Drip	Shallot aphid	*Myzus ascalonicus*	Hexapoda	Hemiptera	Aphididae
ABW96354	Drip	Sweet potato whitefly	*Bemisia tabaci*	Hexapoda	Hemiptera	Aleyrodidae
Q23808	Drip	Green leafhopper	*Cicadella viridis*	Hexapoda	Hemiptera	Cicadellidae
GAGF01044362	Drip	Green lacewing	*Chrysopa pallens*	Hexapoda	Neoptera	Chrysopidae
JPZV01194828	Drip	German cockroach	*Blattella germanica*	Hexapoda	Blattodea	Ectobiidae
GAWS01023831	Drip	American cockroach	*Periplaneta americana*	Hexapoda	Blattodea	Blattidae
GAYD01018072	Drip	Blaberus cockroach	*Blaberus atropos*	Hexapoda	Blattodea	Blaberidae
GAZN01021910	Drip	Brown hooded cockroach	*Cryptocercus wrighti*	Hexapoda	Blattodea	Cryptocercidae
AUST01002328/AUST01002331/AUST01002332	Drip	Nevada dampwood termite	*Zootermopsis nevadensis*	Hexapoda	Isoptera	Termopsidae
BAG72254	Drip	Formosan subterranean termite	*Coptotermes formosanus*	Hexapoda	Isoptera	Rhinotermitidae
GASE01015407	Drip 1	Cuban subterranean termite	*Prorhinotermes simplex*	Hexapoda	Isoptera	Rhinotermitidae
GASE01015406	Drip 2	Cuban subterranean termite	*Prorhinotermes simplex*	Hexapoda	Isoptera	Rhinotermitidae
FL637621/GO899611	Drip	Eastern subterranean termite	*Reticulitermes flavipes*	Hexapoda	Isoptera	Rhinotermitidae
GATB01242877	Drip	Metallyticid mantis	*Metallyticus splendidus*	Hexapoda	Mantodea	Metallyticidae
GASW01221493	Drip	Praying mantis	*Mantis religiosa*	Hexapoda	Mantodea	Mantidae
GAYA01397859	Drip	Zoraptid	*Zorotypus caudelli*	Hexapoda	Orthoptera	Zorotypidae
GAYQ01184451	Drip	European earwig	*Forficula auricularia*	Hexapoda	Dermaptera	Forficulidae
GAUF01006901	Drip	Leuctra	*Leuctra sp.*	Hexapoda	Plecoptera	Leuctridae
GATV01089684	Drip	Stonefly	*Perla marginata*	Hexapoda	Plecoptera	Perlidae
GAWU01021632	Drip	Webspinner	*Aposthonia japonica*	Hexapoda	Embioptera	Oligotomidae
GAWG01027137	Drip	Giant prickly stick insect	*Extatosoma tiaratum*	Hexapoda	Phasmatodea	Phasmatidae
GAWE01111259	Drip	Vietnamese walking stick insect	*Ramulus artemis*	Hexapoda	Phasmatodea	Phasmatidae
GAWD01031379	Drip	Vietnamese walking stick	*Medauroidea extradentata*	Hexapoda	Phasmatodea	Phasmatidae
GAWC01082932	Drip	Thorny stick insect	*Aretaon asperrimus*	Hexapoda	Phasmatodea	Heteropterygidae
GAXB01016476	Drip	Heelwalker	*Tanzaniophasma sp.*	Hexapoda	Mantophasmatodea	Tanzaniophasmatidae
GAUX01016809	Drip	Camel cricket	*Ceuthophilus sp.*	Hexapoda	Orthoptera	Rhaphidophoridae
GAZT01011867	Drip	False stick insect	*Prosarthria teretrirostris*	Hexapoda	Orthoptera	Proscopiidae
GAIZ01003265/GAIZ01000499	Drip	Sand field cricket	*Gryllus firmus*	Hexapoda	Orthoptera	Gryllidae
GBHB01065040	Drip	Oceanic field cricket	*Teleogryllus commodus*	Hexapoda	Orthoptera	Gryllidae
GASQ01128475	Drip	Slender Groundhopper	*Tetrix subulata*	Hexapoda	Orthoptera	Tetrigidae
JG680985	Drip	Desert locust	*Schistocerca gregaria*	Hexapoda	Orthoptera	Acrididae
CO850494/AVCP010961306	Drip	Migratory locust	*Locusta migratoria*	Hexapoda	Orthoptera	Acrididae
GAUZ01033299	Drip	Stripe-winged grasshopper	*Stenobothrus lineatus*	Hexapoda	Orthoptera	Acrididae
GAXA01100045	Drip	Mahogany Dun	*Isonychia bicolor*	Hexapoda	Ephemeroptera	Isonychiidae
GAZG01016414	Drip	Mayfly	*Eurylophella sp.*	Hexapoda	Ephemeroptera	Ephemerellidae
AYNC01036599/AYNC01036594/AYNC01036593/AYNC01036592	Drip	Green drake	*Ephemera danica*	Hexapoda	Ephemeroptera	Ephemeridae
GAYO01010968	Drip	Golden-ringed dragonfly	*Cordulegaster boltonii*	Hexapoda	Odonata	Cordulegastridae
GAEQ01000128	Drip 1	Hagen´s bluet	*Enallagma hageni*	Hexapoda	Odonata	Coenagrionidae
GAEQ01002752	Drip 2	Hagen´s bluet	*Enallagma hageni*	Hexapoda	Odonata	Coenagrionidae
APVN01111337/APVN01111338/APVN01111340	Drip	Scarce chaser	*Ladona fulva*	Hexapoda	Odonata	Libellulidae
GASN01407843	Drip	Firebrat	*Thermobia domestica*	Hexapoda	Zygentoma	Lepismatidae
GAYJ01029112	Drip	Silverfish	*Atelura formicaria*	Hexapoda	Zygentoma	Nicoletiidae
GASO01258000	Drip	Silverfish	*Tricholepidion gertschi*	Hexapoda	Zygentoma	Libellulidae
GAUM01185038	Drip	Bristletail	*Machilis hrabei*	Hexapoda	Archaeognatha	Machilidae
GAUG01019014	Drip	Bristletail	*Meinertellus cundinamarcensis*	Hexapoda	Archaeognatha	Meinertellidae
GAYN01143457	Drip	Campodea	*Campodea augens*	Hexapoda	Diplura	Campodeidae
GAMM01004264/GAMM01007190	Drip	Springtail	*Orchesella cincta*	Hexapoda	Collembola	Entomobryidae
EV475341/GASX01090561/GAMN01000162	Drip	Springtail	*Folsomia candida*	Hexapoda	Collembola	Isotomidae
sb_006_05H07	Drip -like	Springtail	*Megaphorura arctica*	Hexapoda	Collembola	Onychiuridae
GATZ01010361	Drip -like	Clover springtail	*Sminthurus viridis*	Hexapoda	Collembola	Sminthuridae
GAUE01009798	Drip -like	Cosmopolitan springtail	*Anurida maritima*	Hexapoda	Collembola	Neanuridae
GAXI01003650	Drip -like	Giant springtail	*Tetrodontophora bielanensis*	Hexapoda	Collembola	Onychiuridae
GATD01013001	Drip -like	Springtail	*Pogonognathellus sp.*	Hexapoda	Collembola	Tomoceridae

Arthropoda Prip

FBpp0087236	Prip	Fruit fly	*Drosophila melanogaster*	Hexapoda	Diptera	Drosophilidae
FBpp0202768	Prip	Fruit fly	*Drosophila sechellia*	Hexapoda	Diptera	Drosophilidae
FBpp0209200	Prip	Fruit fly	*Drosophila simulans*	Hexapoda	Diptera	Drosophilidae
FBpp0257373	Prip	Fruit fly	*Drosophila yakuba*	Hexapoda	Diptera	Drosophilidae
FBpp0138749	Prip	Fruit fly	*Drosophila erecta*	Hexapoda	Diptera	Drosophilidae
FBpp0115747	Prip	Fruit fly	*Drosophila ananassae*	Hexapoda	Diptera	Drosophilidae
FBpp0277602	Prip	Fruit fly	*Drosophila pseudoobscura*	Hexapoda	Diptera	Drosophilidae
FBpp0234775	Prip	Fruit fly	*Drosophila virilis*	Hexapoda	Diptera	Drosophilidae
FBpp0167781	Prip	Fruit fly	*Drosophila mojavensis*	Hexapoda	Diptera	Drosophilidae
FBpp0155293	Prip	Fruit fly	*Drosophila grimshawi*	Hexapoda	Diptera	Drosophilidae
FBpp0250560	Prip	Fruit fly	*Drosophila willistoni*	Hexapoda	Diptera	Drosophilidae
ACT34032	Prip	Goldenrod gall fly	*Eurosta solidaginis*	Hexapoda	Diptera	Tephritidae
ADD19396	Prip 1	Tsetse fly	*Glossina morsitans morsitans*	Hexapoda	Diptera	Glossinidae
AFP49895	Prip 2	Tsetse fly	*Glossina morsitans morsitans*	Hexapoda	Diptera	Glossinidae
AAEL003550/XP_001656932	Prip	Yellow fever mosquito	*Aedes aegypti*	Hexapoda	Diptera	Culicidae
CPIJ015700	Prip	Southern house mosquito	*Culex quinquefasciatus*	Hexapoda	Diptera	Culicidae
AGAP008843	Prip	African malaria mosquito	*Anopheles gambiae*	Hexapoda	Diptera	Culicidae
BAF62090	Prip	Sleeping chironomid	*Polypedilum vanderplanki*	Hexapoda	Diptera	Chironomidae
BAK32937/BAK32936/BAK32935/GAAK01006838	Prip 1	Antarctic flightless midge	*Belgica antarctica*	Hexapoda	Diptera	Chironomidae
GAAK01006838	Prip 2	Antarctic flightless midge	*Belgica antarctica*	Hexapoda	Diptera	Chironomidae
NP_001153661	Prip	Domestic silkworm	*Bombyx mori*	Hexapoda	Lepidoptera	Bombycidae
CAEZ01007983	Prip	Postman butterfly	*Heliconius melpomene melpomene*	Hexapoda	Lepidoptera	Nymphalidae
EL599805/EL596094	Prip	Red postman	*Heliconius erato*	Hexapoda	Lepidoptera	Nymphalidae
EHJ66754	Prip	Monarch butterfly	*Danaus plexippus*	Hexapoda	Lepidoptera	Nymphalidae
EZ981212	Prip	African cotton leafworm	*Spodoptera littoralis*	Hexapoda	Lepidoptera	Noctuidae
GAFU01006086	Prip	Beet armyworm	*Spodoptera exigua*	Hexapoda	Lepidoptera	Noctuidae
JP717913	Prip	Asian Swallowtail	*Papilio xuthus*	Hexapoda	Lepidoptera	Papilionidae
GAJS01000346	Prip	striped riceborer	*Chilo suppressalis*	Hexapoda	Lepidoptera	Crambidae
JP612966	Prip	Propertius duskywing	*Erynnis propertius*	Hexapoda	Lepidoptera	Hesperiidae
JO818733	Prip	Tobacco hornworm	*Manduca sexta*	Hexapoda	Lepidoptera	Sphingidae

TC001374	Prip	Red flour Beetle	*Tribolium castaneum*	Hexapoda	Coleoptera	Tenebrionidae
AEE61850	Prip	Mountain pine weevil	*Dendroctonus ponderosae*	Hexapoda	Coleoptera	Curculionidae
JU408944	Prip	Salt marsh beetle	*Pogonus chalceus*	Hexapoda	Coleoptera	Carabidae
AAL09065	Prip	Firefly	*Pyrocoelia rufa*	Hexapoda	Coleoptera	Lampyridae
GAXW01003825	Prip	Antlion	*Euroleon nostras*	Hexapoda	Neoptera	Myrmeleontidae
GAVV01005429	Prip	Green lacewing	*Pseudomallada prasinus*	Hexapoda	Neoptera	Chrysopidae
XP_003394168/XP_003394169	Prip	Buff-tailed bumblebee	*Bombus terrestris*	Hexapoda	Hymenoptera	Apidae
XP_003487537/XP_003487538	Prip	Common eastern bumble bee	*Bombus impatiens*	Hexapoda	Hymenoptera	Apidae
XP_394391	Prip	Honey bee	*Apis mellifera*	Hexapoda	Hymenoptera	Apidae
XP_003701667	Prip	Alfalfa leafcutting bee	*Megachile rotundata*	Hexapoda	Hymenoptera	Megachilidae
GAFR01021571	Prip	Neotropical paper wasp	*Polistes canadensis*	Hexapoda	Hymenoptera	Vespidae
EFN88447	Prip	Jerdon's jumping ant	*Harpegnathos saltator*	Hexapoda	Hymenoptera	Formicidae
EFN72835	Prip	Florida carpenter ant	*Camponotus floridanus*	Hexapoda	Hymenoptera	Formicidae
ACEP_00007751	Prip	Leafcutter ant	*Atta cephalotes*	Hexapoda	Hymenoptera	Formicidae
XP_001607929	Prip	Jewel wasp	*Nasonia vitripennis*	Hexapoda	Hymenoptera	Pteromalidae
ADAO01292418/ADAO01292417/ADAO01292415	Prip	Jewel wasp	*Nasonia giraulti*	Hexapoda	Hymenoptera	Pteromalidae
EEB16742	Prip	Human body louse	*Pediculus humanus corporis*	Hexapoda	Phthiraptera	Pediculidae
GAWR01000697	Eglp	Poultry shaft louse	*Menopon gallinae*	Hexapoda	Phthiraptera	Menoponidae
GAYV01110104	Eglp	Booklice	*Liposcelis bostrychophila*	Hexapoda	Psocoptera	Liposcelidae
GAPT01004386	Eglp	Booklice	*Ectopsocus briggsi*	Hexapoda	Psocoptera	Ectopsocidae
GAXD01020785	Prip	Western flower thrips	*Frankliniella occidentalis*	Hexapoda	Thysanoptera	Thripidae
GAHY01001529	Prip	Assassin bug	*Rhodnius prolixus*	Hexapoda	Hemiptera	Reduviidae
KF048099	Prip	Lygus bug	*Lygus hesperus*	Hexapoda	Hemiptera	Miridae
KF048100	Prip	Lygus bug	*Lygus hesperus*	Hexapoda	Hemiptera	Miridae
HP661479	Prip	Sweet potato whitefly	*Bemisia tabaci*	Hexapoda	Hemiptera	Aleyrodidae
AHB86600	Prip	potato/tomato psyllid	*Bactericera cockerelli*	Hexapoda	Hemiptera	Triozidae
DN195967	Prip	Glassy-winged sharpshooter	*Homalodisca vitripennis*	Hexapoda	Hemiptera	Cicadellidae
GAGF01006829	Prip	Green lacewing	*Chrysopa pallens*	Hexapoda	Neoptera	Chrysopidae
CBY77924/GBID01001247	Prip	German cockroach	*Blattella germanica*	Hexapoda	Blattodea	Ectobiidae
GAWS01258646	Prip	American cockroach	*Periplaneta americana*	Hexapoda	Blattodea	Blattidae
GAYD01029081	Prip	Blaberus cockroach	*Blaberus atropos*	Hexapoda	Blattodea	Blaberidae
GAZN01019011	Prip	Brown hooded cockroach	*Cryptocercus wrighti*	Hexapoda	Blattodea	Cryptocercidae
AUST01002325/AUST01002324	Prip	Nevada dampwood termite	*Zootermopsis nevadensis*	Hexapoda	Isoptera	Termopsidae
JK445261/JK445262	Prip	Formosan subterranean termite	*Coptotermes formosanus*	Hexapoda	Isoptera	Rhinotermitidae
GASE01253679	Prip	Cuban subterranean termite	*Prorhinotermes simplex*	Hexapoda	Isoptera	Rhinotermitidae
FL638365/GO907955	Prip	Eastern subterranean termite	*Reticulitermes flavipes*	Hexapoda	Isoptera	Rhinotermitidae
GATB01001403	Prip	Metallyticid mantis	*Metallyticus splendidus*	Hexapoda	Mantodea	Metallyticidae
GASW01019624	Prip	Praying mantis	*Mantis religiosa*	Hexapoda	Mantodea	Mantidae
GAYQ01017598	Prip	European earwig	*Forficula auricularia*	Hexapoda	Dermaptera	Forficulidae
GAYL01013696	Prip	Stonefly	*Cosmioperla kuna*	Hexapoda	Plecoptera	Eustheniidae
GAUF01007405	Prip	Leuctra	*Leuctra sp.*	Hexapoda	Plecoptera	Leuctridae
GAYL01013696	Prip	Stonefly	*Perla marginata*	Hexapoda	Plecoptera	Perlidae
GAWU01256075	Prip	Webspinner	*Aposthonia japonica*	Hexapoda	Embioptera	Oligotomidae
GAWG01042575	Prip	Giant prickly stick insect	*Extatosoma tiaratum*	Hexapoda	Phasmatodea	Phasmatidae
GAWE01078541	Prip	Vietnamese walking stick insect	*Ramulus artemis*	Hexapoda	Phasmatodea	Phasmatidae
GAWD01074919	Prip	Vietnamese walking stick	*Medauroidea extradentata*	Hexapoda	Phasmatodea	Phasmatidae
GAWF01050038/GAWF01050037	Prip	Pink winged stick insect	*Sipyloidea sipylus*	Hexapoda	Phasmatodea	Diapheromeridae
GAWC01082955	Prip	Thorny stick insect	*Aretaon asperrimus*	Hexapoda	Phasmatodea	Heteropterygidae
GAXB01016385	Prip	Heelwalker	*Tanzaniophasma sp.*	Hexapoda	Mantophasmatodea	Tanzaniophasmatidae
GAUX01030554	Prip	Camel cricket	*Ceuthophilus sp.*	Hexapoda	Orthoptera	Rhaphidophoridae
GAZT01006221	Prip	False stick insect	*Prosarthria teretrirostris*	Hexapoda	Orthoptera	Proscopiidae
GAIZ01018549	Prip	Sand field cricket	*Gryllus firmus*	Hexapoda	Orthoptera	Gryllidae
DC443641	Prip	Two-spotted cricket	*Gryllus bimaculatus*	Hexapoda	Orthoptera	Gryllidae
EH632028/EH639574	Prip	Hawaiian swordtail cricket	*Laupala kohalensis*	Hexapoda	Orthoptera	Gryllidae
GBHB01027253	Prip	Oceanic field cricket	*Teleogryllus commodus*	Hexapoda	Orthoptera	Gryllidae
GASQ01011894	Prip	Slender Groundhopper	*Tetrix subulata*	Hexapoda	Orthoptera	Tetrigidae
CO821055/CO821056	Prip	Migratory locust	*Locusta migratoria*	Hexapoda	Orthoptera	Acrididae
GAUZ01034179	Prip	Stripe-winged grasshopper	*Stenobothrus lineatus*	Hexapoda	Orthoptera	Acrididae
GATU01010497	Prip	Blue-winged olive	*Baetis sp.*	Hexapoda	Ephemeroptera	Baetidae
GAXA01011140	Prip	Mahogany Dun	*Isonychia bicolor*	Hexapoda	Ephemeroptera	Isonychiidae
GAZG01016497	Prip	Mayfly	*Eurylophella sp.*	Hexapoda	Ephemeroptera	Ephemerellidae
AYNC01078222	Prip	Green drake	*Ephemera danica*	Hexapoda	Ephemeroptera	Ephemeridae
GAYM01100186	Prip	Banded damoiselle	*Calopteryx splendens*	Hexapoda	Odonata	Calopterygidae
GAVW01127800	Prip	Dragonfly	*Epiophlebia superstes*	Hexapoda	Odonata	Epiophlebiidae
GAYO01130920	Prip 1	Golden-ringed dragonfly	*Cordulegaster boltonii*	Hexapoda	Odonata	Cordulegastridae
GAYO01011149	Prip 2	Golden-ringed dragonfly	*Cordulegaster boltonii*	Hexapoda	Odonata	Cordulegastridae
APVN01148320	Prip 1	Scarce chaser	*Ladona fulva*	Hexapoda	Odonata	Libellulidae
APVN01148314/APVN01148315	Prip 2	Scarce chaser	*Ladona fulva*	Hexapoda	Odonata	Libellulidae
APVN01148320/APVN01148322	Prip 3	Scarce chaser	*Ladona fulva*	Hexapoda	Odonata	Libellulidae
APVN01148322	Prip 4	Scarce chaser	*Ladona fulva*	Hexapoda	Odonata	Libellulidae
GASN01409111	Prip	Firebrat	*Thermobia domestica*	Hexapoda	Zygentoma	Lepismatidae
GAYJ01029739	Prip	Silverfish	*Atelura formicaria*	Hexapoda	Zygentoma	Nicoletiidae
GASO01021146	Prip	Silverfish	*Tricholepidion gertschi*	Hexapoda	Zygentoma	Libellulidae
FN222978	Prip	Bristletail	*Lepismachilis y-signata*	Hexapoda	Archaeognatha	Machilidae
GAUM01020136	Prip	Bristletail	*Machilis hrabei*	Hexapoda	Archaeognatha	Machilidae
GAUG01033311	Prip	Bristletail	*Meinertellus cundinamarcensis*	Hexapoda	Archaeognatha	Meinertellidae
GAYN01007080	Prip 1	Campodea	*Campodea augens*	Hexapoda	Diplura	Campodeidae
GAYN01144154	Prip 2	Campodea	*Campodea augens*	Hexapoda	Diplura	Campodeidae
GAXJ01007613	Prip 1	Two-pronged bristletail	*Occasjapyx japonicus*	Hexapoda	Diplura	Japygidae
GAXJ01010312	Prip 2	Two-pronged bristletail	*Occasjapyx japonicus*	Hexapoda	Diplura	Japygidae
GAXJ01010770	Prip 3	Two-pronged bristletail	*Occasjapyx japonicus*	Hexapoda	Diplura	Japygidae
GAXJ01101258	Prip 4	Two-pronged bristletail	*Occasjapyx japonicus*	Hexapoda	Diplura	Japygidae
GAMM01012413/GAMM01012414	Prip -like1	Springtail	*Orchesella cincta*	Hexapoda	Collembola	Entomobryidae
GAMM01010822	Prip -like2	Springtail	*Orchesella cincta*	Hexapoda	Collembola	Entomobryidae
GAMM01009287	Prip -like3	Springtail	*Orchesella cincta*	Hexapoda	Collembola	Entomobryidae
GAMN01008708	Prip -like1	Springtail	*Folsomia candida*	Hexapoda	Collembola	Isotomidae
GAMN01012115/GAMN01012753	Prip -like2	Springtail	*Folsomia candida*	Hexapoda	Collembola	Isotomidae
sb_005_09I19	Prip -like1	Springtail	*Megaphorura arctica*	Hexapoda	Collembola	Onychiuridae
EW748629	Prip -like2	Springtail	*Megaphorura arctica*	Hexapoda	Collembola	Onychiuridae
GATZ01003502	Prip -like1	Clover springtail	*Sminthurus viridis*	Hexapoda	Collembola	Sminthuridae
GATZ01016023	Prip -like2	Clover springtail	*Sminthurus viridis*	Hexapoda	Collembola	Sminthuridae
GATZ01103627	Prip -like3	Clover springtail	*Sminthurus viridis*	Hexapoda	Collembola	Sminthuridae
GAUE01008782	Prip -like1	Cosmopolitan springtail	*Anurida maritima*	Hexapoda	Collembola	Neanuridae
GAUE01009121	Prip -like2	Cosmopolitan springtail	*Anurida maritima*	Hexapoda	Collembola	Neanuridae
GAUE01052561	Prip -like3	Cosmopolitan springtail	*Anurida maritima*	Hexapoda	Collembola	Neanuridae
GAXI01003818	Prip -like1	Giant springtail	*Tetrodontophora bielanensis*	Hexapoda	Collembola	Onychiuridae
GAXI01003820	Prip -like2	Giant springtail	*Tetrodontophora bielanensis*	Hexapoda	Collembola	Onychiuridae
GAXI01020146	Prip -like3	Giant springtail	*Tetrodontophora bielanensis*	Hexapoda	Collembola	Onychiuridae
GATD01009723	Prip -like1	Springtail	*Pogonognathellus sp.*	Hexapoda	Collembola	Tomoceridae

GATD01010900	Prip -like2	Springtail	*Pogonognathellus sp.*	Hexapoda	Collembola	Tomoceridae
EV474850/EV478034	Prip -like V-pa	Springtail	*Folsomia candida*	Hexapoda	Collembola	Isotomidae
GATZ01102432	Prip -like I-par	Clover springtail	*Sminthurus viridis*	Hexapoda	Collembola	Sminthuridae
GAUE01011057	Prip -like I-par	Cosmopolitan springtail	*Anurida maritima*	Hexapoda	Collembola	Neanuridae
GAXI01015403	Prip -like V-pa	Giant springtail	*Tetrodontophora bielanensis*	Hexapoda	Collembola	Onychiuridae
GAXI01018099	Prip -like I-par	Giant springtail	*Tetrodontophora bielanensis*	Hexapoda	Collembola	Onychiuridae
GATD01099700	Prip -like I-par	Springtail	*Pogonognathellus sp.*	Hexapoda	Collembola	Tomoceridae
GAXE01021065	Prip -like1	Conehead	*Acerentomon sp.*	Hexapoda	Protura	Acerentomidae
GAXE01011432	Prip -like2	Conehead	*Acerentomon sp.*	Hexapoda	Protura	Acerentomidae
AFFK01018906	Prip -like	Coastal European centipede	*Strigamia maritima*	Chilopoda	Geophilomorpha	Linotaeniidae
AFFK01016279	Prip -like	Coastal European centipede	*Strigamia maritima*	Chilopoda	Geophilomorpha	Linotaeniidae
AFFK01018906	Prip -like	Coastal European centipede	*Strigamia maritima*	Chilopoda	Geophilomorpha	Linotaeniidae
AFFK01022706	Prip -like	Coastal European centipede	*Strigamia maritima*	Chilopoda	Geophilomorpha	Linotaeniidae
AFFK01021957	Prip -like	Coastal European centipede	*Strigamia maritima*	Chilopoda	Geophilomorpha	Linotaeniidae
AFFK01015980	Prip -like	Coastal European centipede	*Strigamia maritima*	Chilopoda	Geophilomorpha	Linotaeniidae
AFFK01023535/AFFK01023534	Prip -like	Coastal European centipede	*Strigamia maritima*	Chilopoda	Geophilomorpha	Linotaeniidae
AFFK01013572	Prip -like	Coastal European centipede	*Strigamia maritima*	Chilopoda	Geophilomorpha	Linotaeniidae
AFFK01013572	Prip -like	Coastal European centipede	*Strigamia maritima*	Chilopoda	Geophilomorpha	Linotaeniidae
AFFK01013573	Prip -like	Coastal European centipede	*Strigamia maritima*	Chilopoda	Geophilomorpha	Linotaeniidae
AFFK01013573	Prip -like	Coastal European centipede	*Strigamia maritima*	Chilopoda	Geophilomorpha	Linotaeniidae
GAFS01003350	Prip -like	Narrow-clawed crayfish	*Pontastacus leptodactylus*	Malacostraca	Decapoda	Astacidae
AET34919	Prip -like	Giant freshwater prawn	*Macrobrachium rosenbergii*	Malacostraca	Decapoda	Palaemonidae
AEI25531	Prip -like	Black tiger shrimp	*Penaeus monodon*	Malacostraca	Decapoda	Penaeidae
FE136953/JP420962/FE081160	Prip -like	Pacific white shrimp	*Litopenaeus vannamei*	Malacostraca	Decapoda	Penaeidae
FD699313	Prip -like	American lobster	*Homarus americanus*	Malacostraca	Decapoda	Nephropidae
JQ970426	Prip -like	Blue crab	*Callinectes sapidus*	Malacostraca	Decapoda	Portunidae
GT562677	Prip -like	Swimming crab	*Portunus trituberculatus*	Malacostraca	Decapoda	Portunidae
GAKD01017822	Prip -like	Amphipod	*Melita plumulosa*	Malacostraca	Amphipoda	Melitidae
JL195955	Prip -like	Remipede	*Speleonectes cf. tulumensis*	Remipedia	Nectiopoda	Speleonectidae
ACO10737	Prip -like	Caligid copepod	*Caligus rogercresseyi*	Maxillopoda	Siphonostomatoida	Caligidae
GO416492	Prip -like	Cod worm	*Lernaeocera branchialis*	Maxillopoda	Siphonostomatoida	Pennellidae
JV192099	Prip -like	Tide pool copepod	*Tigriopus californicus*	Maxillopoda	Harpacticoida	Harpacticidae
EFX74648	Prip -like	Common water flea	*Daphnia pulex*	Branchiopoda	Diplostraca	Daphniidae
GACK01000433	Prip -like	Zebra tick	*Rhipicephalus pulchellus*	Acari	Ixodida	Ixodidae
ADDG01009295/ADDG01028715	Prip -like	Honeybee mite	*Varroa destructor*	Acari	Mesostigmata	Varroidae
XP_003740496	Prip -like	Western predatory mite	*Metaseiulus occidentalis*	Acari	Mesostigmata	Phytoseiidae
JR695181	Prip -like	Two-spotted spider mite	*Tetranychus urticae*	Acari	Acariformes	Tetranychidae
JT045087	Prip -like1	African social eresid spider	*Stegodyphus tentoriicola*	Arachnida	Araneae	Eresidae
GAZR01012136/JT037476	Prip -like1	African social eresid spider	*Stegodyphus mimosarum*	Arachnida	Araneae	Eresidae
AOMJ01121993	Prip -like1	Common house spider	*Parasteatoda tepidariorum*	Arachnida	Araneae	Theridiidae
GAZS01059885	Prip -like1	Brazilian giant whiteknee tarantula	*Acanthoscurria geniculata*	Arachnida	Araneae	Theraphosidae
JAA92966	Prip -like2	American wandering spider	*Cupiennius salei*	Arachnida	Araneae	Theridiidae
GANL01009509	Prip -like2	Black widow	*Latrodectus tredecimguttatus*	Arachnida	Araneae	Theridiidae
GBCS01013355	Prip -like2	Western black widow	*Latrodectus hesperus*	Arachnida	Araneae	Theridiidae
AOMJ01225662/AOMJ01225654	Prip -like2	Common house spider	*Parasteatoda tepidariorum*	Arachnida	Araneae	Theridiidae
DR443206	Prip -like2	São paulo black tarantula	*Acanthoscurria gomesiana*	Arachnida	Araneae	Theraphosidae
GAZS01036184	Prip -like2	Brazilian giant whiteknee tarantula	*Acanthoscurria geniculata*	Arachnida	Araneae	Theraphosidae
AYEL01066910/AYEL01075601	Prip -like1	Chinese scorpion	*Mesobuthus martensii*	Arachnida	Scorpiones	Buthidae
AXZI01136975/AYEL01075601	Prip -like1	Baja California bark scorpion	*Centruroides exilicauda*	Arachnida	Scorpiones	Buthidae
AYEL01052592	Prip -like2	Chinese scorpion	*Mesobuthus martensii*	Arachnida	Scorpiones	Buthidae
AXZI01008908	Prip -like2	Baja California bark scorpion	*Centruroides exilicauda*	Arachnida	Scorpiones	Buthidae
AYEL01088164	Prip -like3	Chinese scorpion	*Mesobuthus martensii*	Arachnida	Scorpiones	Buthidae
AXZI01127083	Prip -like3	Baja California bark scorpion	*Centruroides exilicauda*	Arachnida	Scorpiones	Buthidae

Hexapoda unclassified

FBpp0072014	Eglp 4	Fruit fly	*Drosophila melanogaster*	Hexapoda	Diptera	Drosophilidae
FBpp0196999	Eglp 4	Fruit fly	*Drosophila sechellia*	Hexapoda	Diptera	Drosophilidae
FBpp0223428	Eglp 4	Fruit fly	*Drosophila simulans*	Hexapoda	Diptera	Drosophilidae
FBpp0256556	Eglp 4	Fruit fly	*Drosophila yakuba*	Hexapoda	Diptera	Drosophilidae
FBpp0138556	Eglp 4	Fruit fly	*Drosophila erecta*	Hexapoda	Diptera	Drosophilidae
FBpp0115022	Eglp 4	Fruit fly	*Drosophila ananassae*	Hexapoda	Diptera	Drosophilidae
FBpp0278011	Eglp 4	Fruit fly	*Drosophila pseudoobscura*	Hexapoda	Diptera	Drosophilidae
FBpp0181703	Eglp 4	Fruit fly	*Drosophila persimilis*	Hexapoda	Diptera	Drosophilidae
FBpp0236772	Eglp 4	Fruit fly	*Drosophila virilis*	Hexapoda	Diptera	Drosophilidae
FBpp0169721	Eglp 4	Fruit fly	*Drosophila mojavensis*	Hexapoda	Diptera	Drosophilidae
FBpp0155125	Eglp 4	Fruit fly	*Drosophila grimshawi*	Hexapoda	Diptera	Drosophilidae
FBpp0252317	Eglp 4	Fruit fly	*Drosophila willistoni*	Hexapoda	Diptera	Drosophilidae
EZ127051	Eglp 4	Apple maggot	*Rhagoletis pomonella*	Hexapoda	Diptera	Tephritidae
GAKB01002522	Eglp 4a	Olive fruit fly	*Bactrocera oleae*	Hexapoda	Diptera	Tephritidae
GAKB01004245	Eglp 4b	Olive fruit fly	*Bactrocera oleae*	Hexapoda	Diptera	Tephritidae
GAKB01003899	Eglp 4c	Olive fruit fly	*Bactrocera oleae*	Hexapoda	Diptera	Tephritidae
ACT34033	Eglp 4	Goldenrod gall fly	*Eurosta solidaginis*	Hexapoda	Diptera	Tephritidae
XM_004522562	Eglp 4	Mediterranean fruit fly	*Ceratitis capitata*	Hexapoda	Diptera	Tephritidae
JG419371	Eglp 4	Australian sheep blowfly	*Lucilia cuprina*	Hexapoda	Diptera	Calliphoridae
FG295968/FG298919	Eglp 4	Primary screw-worm	*Cochliomyia hominivorax*	Hexapoda	Diptera	Calliphoridae
EZ597305	Eglp 4	Flesh fly	*Sarcophaga crassipalpis*	Hexapoda	Diptera	Sarcophagidae
FD460504	Eglp 4	Horn fly	*Haematobia irritans irritans*	Hexapoda	Diptera	Muscidae
AQPM01070455/XP_005183005	Eglp 4a	House fly	*Musca domestica*	Hexapoda	Diptera	Muscidae
AQPM01070459	Eglp 4b	House fly	*Musca domestica*	Hexapoda	Diptera	Muscidae
FBpp0072015	Eglp 3	Fruit fly	*Drosophila melanogaster*	Hexapoda	Diptera	Drosophilidae
FBpp0197000	Eglp 3	Fruit fly	*Drosophila sechellia*	Hexapoda	Diptera	Drosophilidae
FBpp0223429	Eglp 3	Fruit fly	*Drosophila simulans*	Hexapoda	Diptera	Drosophilidae
FBpp0256558	Eglp 3	Fruit fly	*Drosophila yakuba*	Hexapoda	Diptera	Drosophilidae
FBpp0138557	Eglp 3	Fruit fly	*Drosophila erecta*	Hexapoda	Diptera	Drosophilidae
FBpp0115023	Eglp 3	Fruit fly	*Drosophila ananassae*	Hexapoda	Diptera	Drosophilidae
FBpp0278012	Eglp 3	Fruit fly	*Drosophila pseudoobscura*	Hexapoda	Diptera	Drosophilidae
FBpp0181704	Eglp 3	Fruit fly	*Drosophila persimilis*	Hexapoda	Diptera	Drosophilidae
FBpp0169722	Eglp 3	Fruit fly	*Drosophila mojavensis*	Hexapoda	Diptera	Drosophilidae
FBpp0155126	Eglp 3	Fruit fly	*Drosophila grimshawi*	Hexapoda	Diptera	Drosophilidae
FBpp0252320	Eglp 3	Fruit fly	*Drosophila willistoni*	Hexapoda	Diptera	Drosophilidae
FBpp0072016	Eglp 2	Fruit fly	*Drosophila melanogaster*	Hexapoda	Diptera	Drosophilidae
FBpp0197001	Eglp 2	Fruit fly	*Drosophila sechellia*	Hexapoda	Diptera	Drosophilidae
FBpp0223430	Eglp 2	Fruit fly	*Drosophila simulans*	Hexapoda	Diptera	Drosophilidae
FBpp0256559	Eglp 2	Fruit fly	*Drosophila yakuba*	Hexapoda	Diptera	Drosophilidae
FBpp0138558	Eglp 2	Fruit fly	*Drosophila erecta*	Hexapoda	Diptera	Drosophilidae
FBpp0115024	Eglp 2	Fruit fly	*Drosophila ananassae*	Hexapoda	Diptera	Drosophilidae
FBpp0278013	Eglp 2	Fruit fly	*Drosophila pseudoobscura*	Hexapoda	Diptera	Drosophilidae
FBpp0181705	Eglp 2	Fruit fly	*Drosophila persimilis*	Hexapoda	Diptera	Drosophilidae
FBpp0236774	Eglp 2	Fruit fly	*Drosophila virilis*	Hexapoda	Diptera	Drosophilidae

FBpp0155127	Eglp 2	Fruit fly	*Drosophila grimshawi*	Hexapoda	Diptera	Drosophilidae
FBpp0252321	Eglp 2	Fruit fly	*Drosophila willistoni*	Hexapoda	Diptera	Drosophilidae
AFP49896	Eglp 2a	Tsetse fly	*Glossina morsitans morsitans*	Hexapoda	Diptera	Glossinidae
ADD19418	Eglp 2b	Tsetse fly	*Glossina morsitans morsitans*	Hexapoda	Diptera	Glossinidae
AFP49898	Eglp 2c	Tsetse fly	*Glossina morsitans morsitans*	Hexapoda	Diptera	Glossinidae
ADD18960	Eglp 2d	Tsetse fly	*Glossina morsitans morsitans*	Hexapoda	Diptera	Glossinidae
AAEL005008/XP_001650169	Eglp 2	Yellow fever mosquito	*Aedes aegypti*	Hexapoda	Diptera	Culicidae
JO854615	Eglp 2	Asian tiger mosquito	*Aedes albopictus*	Hexapoda	Diptera	Culicidae
CPIJ009225	Eglp 2	Southern house mosquito	*Culex quinquefasciatus*	Hexapoda	Diptera	Culicidae
AGAP010325	Eglp 2	African malaria mosquito	*Anopheles gambiae*	Hexapoda	Diptera	Culicidae
EZ975669	Eglp 2	African malaria mosquito	*Anopheles funestus*	Hexapoda	Diptera	Culicidae
GAFE01000152	Eglp 2	Indonesian malaria mosquito	*Anopheles sinensis*	Hexapoda	Diptera	Culicidae
GAMD01001135	Eglp 2	South American malaria mosquito	*Anopheles aquasalis*	Hexapoda	Diptera	Culicidae
BAF62091	Eglp 2	Sleeping chironomid	*Polypedilum vanderplanki*	Hexapoda	Diptera	Chironomidae
GAAK01006050	Eglp 2	Antarctic flightless midge	*Belgica antarctica*	Hexapoda	Diptera	Chironomidae
KA181130	Eglp 2	Harlequin fly	*Chironomus riparius*	Hexapoda	Diptera	Chironomidae
AEGA01025736	Eglp 2	Hessian fly	*Mayetiola destructor*	Hexapoda	Diptera	Cecidomyiidae
EZ406461	Eglp 2	Hessian fly	*Mayetiola destructor*	Hexapoda	Diptera	Cecidomyiidae
JP552203	Eglp 2	Sand fly	*Phlebotomus papatasi*	Hexapoda	Diptera	Psychodidae
FBpp0071980	Eglp 1	Fruit fly	*Drosophila melanogaster*	Hexapoda	Diptera	Drosophilidae
FBpp0259319	Eglp 1	Fruit fly	*Drosophila yakuba*	Hexapoda	Diptera	Drosophilidae
FBpp0141417	Eglp 1	Fruit fly	*Drosophila erecta*	Hexapoda	Diptera	Drosophilidae
FBpp0116260	Eglp 1	Fruit fly	*Drosophila ananassae*	Hexapoda	Diptera	Drosophilidae
FBpp0281368	Eglp 1	Fruit fly	*Drosophila pseudoobscura*	Hexapoda	Diptera	Drosophilidae
FBpp0185590	Eglp 1	Fruit fly	*Drosophila persimilis*	Hexapoda	Diptera	Drosophilidae
FBpp0234409	Eglp 1a	Fruit fly	*Drosophila virilis*	Hexapoda	Diptera	Drosophilidae
FBpp0168243	Eglp 1a	Fruit fly	*Drosophila mojavensis*	Hexapoda	Diptera	Drosophilidae
FBpp0154569	Eglp 1a	Fruit fly	*Drosophila grimshawi*	Hexapoda	Diptera	Drosophilidae
FBpp0252206	Eglp 1	Fruit fly	*Drosophila willistoni*	Hexapoda	Diptera	Drosophilidae
AAEL005001/XP_001650168	Eglp 1	Yellow fever mosquito	*Aedes aegypti*	Hexapoda	Diptera	Culicidae
JO889430	Eglp 1	Asian tiger mosquito	*Aedes albopictus*	Hexapoda	Diptera	Culicidae
CPIJ009224	Eglp 1	Southern house mosquito	*Culex quinquefasciatus*	Hexapoda	Diptera	Culicidae
AGAP010326	Eglp 1	African malaria mosquito	*Anopheles gambiae*	Hexapoda	Diptera	Culicidae
JP541300	Eglp 1	Sand fly	*Phlebotomus papatasi*	Hexapoda	Diptera	Psychodidae
GAAK01006051	Eglp 1a	Antarctic flightless midge	*Belgica antarctica*	Hexapoda	Diptera	Chironomidae
GAAK01006051	Eglp 1b	Antarctic flightless midge	*Belgica antarctica*	Hexapoda	Diptera	Chironomidae
AK383767	Eglp 1	Domestic silkworm	*Bombyx mori*	Hexapoda	Lepidoptera	Bombycidae
AB245966	Eglp 2	Domestic silkworm	*Bombyx mori*	Hexapoda	Lepidoptera	Bombycidae
CAEZ01006548	Eglp 1	Postman butterfly	*Heliconius melpomene melpomene*	Hexapoda	Lepidoptera	Nymphalidae
CAEZ01001062	Eglp 2	Postman butterfly	*Heliconius melpomene melpomene*	Hexapoda	Lepidoptera	Nymphalidae
CAEZ01001062	Eglp 3	Postman butterfly	*Heliconius melpomene melpomene*	Hexapoda	Lepidoptera	Nymphalidae
CAEZ01001062	Eglp 4	Postman butterfly	*Heliconius melpomene melpomene*	Hexapoda	Lepidoptera	Nymphalidae
EHJ72288	Eglp 1	Monarch butterfly	*Danaus plexippus*	Hexapoda	Lepidoptera	Nymphalidae
EHJ65755	Eglp 2	Monarch butterfly	*Danaus plexippus*	Hexapoda	Lepidoptera	Nymphalidae
EHJ78266	Eglp 3	Monarch butterfly	*Danaus plexippus*	Hexapoda	Lepidoptera	Nymphalidae
EHJ72289	Eglp 4	Monarch butterfly	*Danaus plexippus*	Hexapoda	Lepidoptera	Nymphalidae
EB823882	Eglp 2	Indianmeal moth	*Plodia interpunctella*	Hexapoda	Lepidoptera	Pyralidae
JP717914	Eglp 2	Asian Swallowtail	*Papilio xuthus*	Hexapoda	Lepidoptera	Papilionidae
GAJS01025088	Eglp 1	striped riceborer	*Chilo suppressalis*	Hexapoda	Lepidoptera	Crambidae
GAJS01070620	Eglp 2	striped riceborer	*Chilo suppressalis*	Hexapoda	Lepidoptera	Crambidae
JP612965	Eglp 2	Propertius duskywing	*Erynnis propertius*	Hexapoda	Lepidoptera	Hesperiidae
JO814913	Eglp 1	Tobacco hornworm	*Manduca sexta*	Hexapoda	Lepidoptera	Sphingidae
BAH47555	Eglp 1	Oriental fruit moth	*Grapholita molesta*	Hexapoda	Lepidoptera	Tortricidae
TC014278	Eglp 4	Red flour Beetle	*Tribolium castaneum*	Hexapoda	Coleoptera	Tenebrionidae
TC014279	Eglp 3	Red flour Beetle	*Tribolium castaneum*	Hexapoda	Coleoptera	Tenebrionidae
TC014280	Eglp 2	Red flour Beetle	*Tribolium castaneum*	Hexapoda	Coleoptera	Tenebrionidae
TC014281	Eglp 1	Red flour Beetle	*Tribolium castaneum*	Hexapoda	Coleoptera	Tenebrionidae
GAFI01017518	Eglp 1	Southern pine beetle	*Dendroctonus frontalis*	Hexapoda	Coleoptera	Curculionidae
GACR01006542	Eglp 1	European spruce bark beetle	*Ips typographus*	Hexapoda	Coleoptera	Curculionidae
AEE61512	Eglp 1	Mountain pine weevil	*Dendroctonus ponderosae*	Hexapoda	Coleoptera	Curculionidae
JU408487	Eglp 1	Salt marsh beetle	*Pogonus chalceus*	Hexapoda	Coleoptera	Carabidae
GAXW01009218	Eglp	Antlion	*Euroleon nostras*	Hexapoda	Neoptera	Myrmeleontidae
GAVV01181849	Eglp 1	Green lacewing	*Pseudomallada prasinus*	Hexapoda	Neoptera	Chrysopidae
GAVV01166415	Eglp 2	Green lacewing	*Pseudomallada prasinus*	Hexapoda	Neoptera	Chrysopidae
XP_003403182	Eglp 3	Buff-tailed bumblebee	*Bombus terrestris*	Hexapoda	Hymenoptera	Apidae
XP_003486426	Eglp 3	Common eastern bumble bee	*Bombus impatiens*	Hexapoda	Hymenoptera	Apidae
XP_624194	Eglp 3	Honey bee	*Apis mellifera*	Hexapoda	Hymenoptera	Apidae
GAGH01067111	Eglp 3	Mason bee	*Osmia cornuta*	Hexapoda	Hymenoptera	Megachilidae
XP_003702537	Eglp 3	Alfalfa leafcutting bee	*Megachile rotundata*	Hexapoda	Hymenoptera	Megachilidae
EGI59562	Eglp 3	Panamanian leafcutter ant	*Acromyrmex echinatior*	Hexapoda	Hymenoptera	Formicidae
EFN68166	Eglp 3	Florida carpenter ant	*Camponotus floridanus*	Hexapoda	Hymenoptera	Formicidae
EFN76752	Eglp 3	Jerdon's jumping ant	*Harpegnathos saltator*	Hexapoda	Hymenoptera	Formicidae
ACEP_00012182	Eglp 3	Leafcutter ant	*Atta cephalotes*	Hexapoda	Hymenoptera	Formicidae
XP_001601253	Eglp 3	Jewel wasp	*Nasonia vitripennis*	Hexapoda	Hymenoptera	Pteromalidae
ADAO01239456/ADAO01239457/ADAO01239458	Eglp 3	Jewel wasp	*Nasonia giraulti*	Hexapoda	Hymenoptera	Pteromalidae
XP_003403164	Eglp 2	Buff-tailed bumblebee	*Bombus terrestris*	Hexapoda	Hymenoptera	Apidae
XP_003486425	Eglp 2	Common eastern bumble bee	*Bombus impatiens*	Hexapoda	Hymenoptera	Apidae
XP_001121899	Eglp 2	Honey bee	*Apis mellifera*	Hexapoda	Hymenoptera	Apidae
XP_003702536	Eglp 2	Alfalfa leafcutting bee	*Megachile rotundata*	Hexapoda	Hymenoptera	Megachilidae
EGI59563	Eglp 2	Panamanian leafcutter ant	*Acromyrmex echinatior*	Hexapoda	Hymenoptera	Formicidae
EFZ21244	Eglp 2	Red fire ant	*Solenopsis invicta*	Hexapoda	Hymenoptera	Formicidae
ACEP_00012181	Eglp 2	Leafcutter ant	*Atta cephalotes*	Hexapoda	Hymenoptera	Formicidae
XP_001601231	Eglp 2	Jewel wasp	*Nasonia vitripennis*	Hexapoda	Hymenoptera	Pteromalidae
ADAO01239444	Eglp 2	Jewel wasp	*Nasonia giraulti*	Hexapoda	Hymenoptera	Pteromalidae
XP_003393900	Eglp 1	Buff-tailed bumblebee	*Bombus terrestris*	Hexapoda	Hymenoptera	Apidae
XP_003484555	Eglp 1	Common eastern bumble bee	*Bombus impatiens*	Hexapoda	Hymenoptera	Apidae
XP_001121043	Eglp 1	Honey bee	*Apis mellifera*	Hexapoda	Hymenoptera	Apidae
XP_003700908	Eglp 1	Alfalfa leafcutting bee	*Megachile rotundata*	Hexapoda	Hymenoptera	Megachilidae
EGI63170	Eglp 1	Panamanian leafcutter ant	*Acromyrmex echinatior*	Hexapoda	Hymenoptera	Formicidae
EFN67363	Eglp 1	Florida carpenter ant	*Camponotus floridanus*	Hexapoda	Hymenoptera	Formicidae
XP_001603421	Eglp 1	Jewel wasp	*Nasonia vitripennis*	Hexapoda	Hymenoptera	Pteromalidae
ADAO01181999	Eglp 1	Jewel wasp	*Nasonia giraulti*	Hexapoda	Hymenoptera	Pteromalidae
PHUM474700	Eglp 2	Human body louse	*Pediculus humanus corporis*	Hexapoda	Phthiraptera	Pediculidae
PHUM369010	Eglp 1	Human body louse	*Pediculus humanus corporis*	Hexapoda	Phthiraptera	Pediculidae
GAWR01092467	Eglp 1	Poultry shaft louse	*Menopon gallinae*	Hexapoda	Phthiraptera	Menoponidae
GAWR01093370	Eglp 2	Poultry shaft louse	*Menopon gallinae*	Hexapoda	Phthiraptera	Menoponidae
GAWR01009252	Eglp 3	Poultry shaft louse	*Menopon gallinae*	Hexapoda	Phthiraptera	Menoponidae
GAWR01011369	Eglp 4	Poultry shaft louse	*Menopon gallinae*	Hexapoda	Phthiraptera	Menoponidae
GAYV01019679	Eglp 1	Booklice	*Liposcelis bostrychophila*	Hexapoda	Psocoptera	Liposcelidae

GAYV01019146	Eglp 2	Booklice	*Liposcelis bostrychophila*	Hexapoda	Psocoptera	Liposcelidae
GAPT01001679	Eglp 1	Booklice	*Ectopsocus briggsi*	Hexapoda	Psocoptera	Ectopsocidae
GAPT01090202	Eglp 2	Booklice	*Ectopsocus briggsi*	Hexapoda	Psocoptera	Ectopsocidae
GAXD01000016	Eglp	Western flower thrips	*Frankliniella occidentalis*	Hexapoda	Thysanoptera	Thripidae
GAJY01002108	Eglp	Cowpea flower thrips	*Megalurothrips sjostedti*	Hexapoda	Thysanoptera	Thripidae
CAC13959/AEV57515/AEV57516	Eglp	Assassin bug	*Rhodnius prolixus*	Hexapoda	Hemiptera	Reduviidae
KF048098	Eglp 1	Lygus bug	*Lygus hesperus*	Hexapoda	Hemiptera	Miridae
KF048093	Eglp 2A	Lygus bug	*Lygus hesperus*	Hexapoda	Hemiptera	Miridae
KF048094	Eglp 2B	Lygus bug	*Lygus hesperus*	Hexapoda	Hemiptera	Miridae
KF048095	Eglp 2C	Lygus bug	*Lygus hesperus*	Hexapoda	Hemiptera	Miridae
KF048096	Eglp 2D	Lygus bug	*Lygus hesperus*	Hexapoda	Hemiptera	Miridae
KF048097	Eglp 2E	Lygus bug	*Lygus hesperus*	Hexapoda	Hemiptera	Miridae
XP_001952198	Eglp	Pea aphid	*Acyrthosiphon pisum*	Hexapoda	Hemiptera	Aphididae
FO032683	Eglp	Shallot aphid	*Myzus ascalonicus*	Hexapoda	Hemiptera	Aphididae
GAOM01002305	Eglp	Potato aphid	*Macrosiphum euphorbiae*	Hexapoda	Hemiptera	Aphididae
GW522658	Eglp	Cotton aphid	*Aphis gossypii*	Hexapoda	Hemiptera	Aphididae
AHB86603	Eglp	Potato psyllid	*Bactericera cockerelli*	Hexapoda	Hemiptera	Triozidae
AHB86602	Eglp	Potato psyllid	*Bactericera cockerelli*	Hexapoda	Hemiptera	Triozidae
AHB86601	Eglp	Potato psyllid	*Bactericera cockerelli*	Hexapoda	Hemiptera	Triozidae
XP_008487274	Eglp	Asian citrus psyllid	*Diaphorina citri*	Hexapoda	Hemiptera	Psyllidae
GACJ01000325	Eglp	Asian citrus psyllid	*Diaphorina citri*	Hexapoda	Hemiptera	Psyllidae
HP650016	Eglp 1	Sweet potato whitefly	*Bemisia tabaci*	Hexapoda	Hemiptera	Aleyrodidae
HP660290	Eglp 2	Sweet potato whitefly	*Bemisia tabaci*	Hexapoda	Hemiptera	Aleyrodidae
GAJV01000737	Eglp	Cowpea pod-sucking bug	*Anoplocnemis curvipes*	Hexapoda	Hemiptera	Coreidae
GAJX01000223	Eglp	African pod bug	*Clavigralla tomentosicollis*	Hexapoda	Hemiptera	Coreidae
BAN21211	Eglp	Bean bug	*Riptortus pedestris*	Hexapoda	Hemiptera	Coreidae
GBID01002499	Eglp	German cockroach	*Blattella germanica*	Hexapoda	Blattodea	Ectobiidae
FG131060/GAWS01002503	Eglp	American cockroach	*Periplaneta americana*	Hexapoda	Blattodea	Blattidae
GAYD01331798	Eglp	Blaberus cockroach	*Blaberus atropos*	Hexapoda	Blattodea	Blaberidae
GAZN01202855	Eglp 1	Brown hooded cockroach	*Cryptocercus wrighti*	Hexapoda	Blattodea	Cryptocercidae
GAZN01196580	Eglp 2	Brown hooded cockroach	*Cryptocercus wrighti*	Hexapoda	Blattodea	Cryptocercidae
FX376451	Eglp	Wood-eating higher termite	*Nasutitermes takasagoensis*	Hexapoda	Isoptera	Termitidae
AUST01030857	Eglp	Nevada dampwood termite	*Zootermopsis nevadensis*	Hexapoda	Isoptera	Termopsidae
GASE01009002	Eglp	Cuban subterranean termite	*Prorhinotermes simplex*	Hexapoda	Isoptera	Rhinotermitidae
FL640174/FL639044	Eglp	Eastern subterranean termite	*Reticulitermes flavipes*	Hexapoda	Isoptera	Rhinotermitidae
GATB01335234	Eglp	Metallyticid mantis	*Metallyticus splendidus*	Hexapoda	Mantodea	Metallyticidae
GASW01223743	Eglp	Praying mantis	*Mantis religiosa*	Hexapoda	Mantodea	Mantidae
GAYA01013920	Eglp	Zoraptid	*Zorotypus caudelli*	Hexapoda	Orthoptera	Zorotypidae
GAAX01017048	Eglp	European earwig	*Forficula auricularia*	Hexapoda	Dermaptera	Forficulidae
GAYL01128297	Eglp	Stonefly	*Cosmioperla kuna*	Hexapoda	Plecoptera	Eustheniidae
GAUF01000114	Eglp	Leuctra	*Leuctra sp.*	Hexapoda	Plecoptera	Leuctridae
GATV01154381	Eglp	Stonefly	*Perla marginata*	Hexapoda	Plecoptera	Perlidae
GAWU01009413	Eglp	Webspinner	*Aposthonia japonica*	Hexapoda	Embioptera	Oligotomidae
GAWG01091169	Eglp	Giant prickly stick insect	*Extatosoma tiaratum*	Hexapoda	Phasmatodea	Phasmatidae
GAWE01075831	Eglp	Vietnamese walking stick insect	*Ramulus artemis*	Hexapoda	Phasmatodea	Phasmatidae
GAWD01039255	Eglp	Vietnamese walking stick	*Medauroidea extradentata*	Hexapoda	Phasmatodea	Phasmatidae
GAWF01050690	Eglp	Pink winged stick insect	*Sipyloidea sipylus*	Hexapoda	Phasmatodea	Diapheromeridae
GAWC01056573	Eglp	Thorny stick insect	*Aretaon asperrimus*	Hexapoda	Phasmatodea	Heteropterygidae
GAXB01152638	Eglp 1	Heelwalker	*Tanzaniophasma sp.*	Hexapoda	Mantophasmatodea	Tanzaniophasmatidae
GAXB01151664	Eglp 2	Heelwalker	*Tanzaniophasma sp.*	Hexapoda	Mantophasmatodea	Tanzaniophasmatidae
AVCP010359984	Eglp	Migratory locust	*Locusta migratoria*	Hexapoda	Orthoptera	Acrididae
GAZT01001309	Eglp	False stick insect	*Prosarthria teretrirostris*	Hexapoda	Orthoptera	Proscopiidae
GBHB01072046	Eglp	Oceanic field cricket	*Teleogryllus commodus*	Hexapoda	Orthoptera	Gryllidae
GAIZ01015418	Eglp	Sand field cricket	*Gryllus firmus*	Hexapoda	Orthoptera	Gryllidae
GASQ01128632	Eglp	Slender Groundhopper	*Tetrix subulata*	Hexapoda	Orthoptera	Tetrigidae
GAUZ01005949	Eglp	Stripe-winged grasshopper	*Stenobothrus lineatus*	Hexapoda	Orthoptera	Acrididae
GATU01013392	Eglp 1	Blue-winged olive	*Baetis sp.*	Hexapoda	Ephemeroptera	Baetidae
GATU01003749	Eglp 2	Blue-winged olive	*Baetis sp.*	Hexapoda	Ephemeroptera	Baetidae
GAXA01012395	Eglp	Mahogany Dun	*Isonychia bicolor*	Hexapoda	Ephemeroptera	Isonychiidae
GAZG01100517	Eglp	Mayfly	*Eurylophella sp.*	Hexapoda	Ephemeroptera	Ephemerellidae
AYNC01028369	Eglp	Green drake	*Ephemera danica*	Hexapoda	Ephemeroptera	Ephemeridae
GAYM01007100	Eglp	Banded damoiselle	*Calopteryx splendens*	Hexapoda	Odonata	Calopterygidae
GAVW01007834	Eglp	Dragonfly	*Epiophlebia superstes*	Hexapoda	Odonata	Epiophlebiidae
GAYO01001919	Eglp	Golden-ringed dragonfly	*Cordulegaster boltonii*	Hexapoda	Odonata	Cordulegastridae
APVN01045462	Eglp	Scarce chaser	*Ladona fulva*	Hexapoda	Odonata	Libellulidae
GASN01409637	Eglp	Firebrat	*Thermobia domestica*	Hexapoda	Zygentoma	Lepismatidae
GAYJ01034252	Eglp	Silverfish	*Atelura formicaria*	Hexapoda	Zygentoma	Nicoletiidae
GAUM01182693	Eglp	Bristletail	*Machilis hrabei*	Hexapoda	Archaeognatha	Machilidae
GAUG01034808	Eglp	Bristletail	*Meinertellus cundinamarcensis*	Hexapoda	Archaeognatha	Meinertellidae
GAUM01024706	Eglp -like	Bristletail	*Machilis hrabei*	Hexapoda	Archaeognatha	Machilidae
GAUG01247769	Eglp -like	Bristletail	*Meinertellus cundinamarcensis*	Hexapoda	Archaeognatha	Meinertellidae
GAXE01013530	Eglp -like I-para	Conehead	*Acerentomon sp.*	Hexapoda	Protura	Acerentomidae
GAXE01015084	Eglp -like S-par	Conehead	*Acerentomon sp.*	Hexapoda	Protura	Acerentomidae
**Arthropoda aquaglyceroporins**						
XP_002430403	Glp	Human body louse	*Pediculus humanus corporis*	Hexapoda	Phthiraptera	Pediculidae
GAWR01093456	Glp	Poultry shaft louse	*Menopon gallinae*	Hexapoda	Phthiraptera	Menoponidae
GAYV01015247	Glp 1	Booklice	*Liposcelis bostrychophila*	Hexapoda	Psocoptera	Liposcelidae
GAYV01015248	Glp 2	Booklice	*Liposcelis bostrychophila*	Hexapoda	Psocoptera	Liposcelidae
GAPT01019852	Glp	Booklice	*Ectopsocus briggsi*	Hexapoda	Psocoptera	Ectopsocidae
GAYA01037781	Glp	Zoraptid	*Zorotypus caudelli*	Hexapoda	Orthoptera	Zorotypidae
GABA01000698	Glp	Zoraptid	*Zorotypus gurneyi*	Hexapoda	Zoraptera	Zorotypidae
GAYQ01177068	Glp	European earwig	*Forficula auricularia*	Hexapoda	Dermaptera	Forficulidae
GAYL01081166	Glp	Stonefly	*Cosmioperla kuna*	Hexapoda	Plecoptera	Eustheniidae
GAUF01080011	Glp	Leuctra	*Leuctra sp.*	Hexapoda	Plecoptera	Leuctridae
GATV01110784	Glp	Stonefly	*Perla marginata*	Hexapoda	Plecoptera	Perlidae
GAWU01218234	Glp	Webspinner	*Aposthonia japonica*	Hexapoda	Embioptera	Oligotomidae
GAWG01068929	Glp	Giant prickly stick insect	*Extatosoma tiaratum*	Hexapoda	Phasmatodea	Phasmatidae
GAWE01083837/GAWE01083816	Glp	Vietnamese walking stick insect	*Ramulus artemis*	Hexapoda	Phasmatodea	Phasmatidae
GAWD01050986	Glp	Vietnamese walking stick	*Medauroidea extradentata*	Hexapoda	Phasmatodea	Phasmatidae
GAWF01020399/GAWF01096573/GAWF01024650/ GAWF01024651	Glp	Pink winged stick insect	*Sipyloidea sipylus*	Hexapoda	Phasmatodea	Diapheromeridae
GAWC01056095	Glp	Thorny stick insect	*Aretaon asperrimus*	Hexapoda	Phasmatodea	Heteropterygidae
GAXB01138367	Glp	Heelwalker	*Tanzaniophasma sp.*	Hexapoda	Mantophasmatodea	Tanzaniophasmatidae
GAUX01277991	Glp	Camel cricket	*Ceuthophilus sp.*	Hexapoda	Orthoptera	Rhaphidophoridae
GAZT01142164	Glp	False stick insect	*Prosarthria teretrirostris*	Hexapoda	Orthoptera	Proscopiidae
GASQ01015062	Glp	Slender Groundhopper	*Tetrix subulata*	Hexapoda	Orthoptera	Tetrigidae
GAUZ01006460	Glp	Stripe-winged grasshopper	*Stenobothrus lineatus*	Hexapoda	Orthoptera	Acrididae
GATU01072356	Glp	Blue-winged olive	*Baetis sp.*	Hexapoda	Ephemeroptera	Baetidae
GAXA01113250	Glp	Mahogany Dun	*Isonychia bicolor*	Hexapoda	Ephemeroptera	Isonychiidae

GAZG01101517	Glp	Mayfly	*Eurylophella sp.*	Hexapoda	Ephemeroptera	Ephemerellidae
AYNC01028228/AYNC01028227	Glp	Green drake	*Ephemera danica*	Hexapoda	Ephemeroptera	Ephemeridae
GAYM01097963	Glp	Banded damoiselle	*Calopteryx splendens*	Hexapoda	Odonata	Calopterygidae
GAVW01126922	Glp	Dragonfly	*Epiophlebia superstes*	Hexapoda	Odonata	Epiophlebiidae
GAYO01012540	Glp	Golden-ringed dragonfly	*Cordulegaster boltonii*	Hexapoda	Odonata	Cordulegastridae
APVN01141542/APVN01141543/APVN01141545	Glp	Scarce chaser	*Ladona fulva*	Hexapoda	Odonata	Libellulidae
GASN01018603	Glp 1	Firebrat	*Thermobia domestica*	Hexapoda	Zygentoma	Lepismatidae
GASN01397590	Glp 2	Firebrat	*Thermobia domestica*	Hexapoda	Zygentoma	Lepismatidae
GAYJ01031386	Glp	Silverfish	*Atelura formicaria*	Hexapoda	Zygentoma	Nicoletiidae
GASO01018965	Glp	Silverfish	*Tricholepidion gertschi*	Hexapoda	Zygentoma	Libellulidae
GAUM01020992	Glp	Bristletail	*Machilis hrabei*	Hexapoda	Archaeognatha	Machilidae
GAUG01249683	Glp	Bristletail	*Meinertellus cundinamarcensis*	Hexapoda	Archaeognatha	Meinertellidae
GAYN01134376	Glp 1	Campodea	*Campodea augens*	Hexapoda	Diplura	Campodeidae
GAYN01140084	Glp 2	Campodea	*Campodea augens*	Hexapoda	Diplura	Campodeidae
JT051615	Glp 1	Two-pronged bristletail	*Megajapyx sp.*	Hexapoda	Diplura	Japygidae
GAXJ01112653	Glp 1	Two-pronged bristletail	*Occasjapyx japonicus*	Hexapoda	Diplura	Japygidae
GAXJ01013070	Glp 2	Two-pronged bristletail	*Occasjapyx japonicus*	Hexapoda	Diplura	Japygidae
GATZ01009105	Glp 1	Clover springtail	*Sminthurus viridis*	Hexapoda	Collembola	Sminthuridae
GATZ01103685	Glp 2	Clover springtail	*Sminthurus viridis*	Hexapoda	Collembola	Sminthuridae
GAUE01002450	Glp 1	Cosmopolitan springtail	*Anurida maritima*	Hexapoda	Collembola	Neanuridae
GAUE01007494	Glp 2	Cosmopolitan springtail	*Anurida maritima*	Hexapoda	Collembola	Neanuridae
GAXI01021746	Glp 1	Giant springtail	*Tetrodontophora bielanensis*	Hexapoda	Collembola	Onychiuridae
GAXI01021160	Glp 2	Giant springtail	*Tetrodontophora bielanensis*	Hexapoda	Collembola	Onychiuridae
GAMM01000769	Glp	Springtail	*Orchesella cincta*	Hexapoda	Collembola	Entomobryidae
GAMN01001199	Glp 1	Springtail	*Folsomia candida*	Hexapoda	Collembola	Isotomidae
GAMN01014673	Glp 2	Springtail	*Folsomia candida*	Hexapoda	Collembola	Isotomidae
GATD01099253	Glp	Springtail	*Pogonognathellus sp.*	Hexapoda	Collembola	Tomoceridae
GAXE01023694	Glp	Conehead	*Acerentomon sp.*	Hexapoda	Protura	Acerentomidae
AFFK01019422/SMAR004742	Glp	Coastal European centipede	*Strigamia maritima*	Chilopoda	Geophilomorpha	Linotaeniidae
GAFS01005819	Glp	Narrow-clawed crayfish	*Pontastacus leptodactylus*	Malacostraca	Decapoda	Astacidae
JP360962/JP360767	Glp	Pacific white shrimp	*Litopenaeus vannamei*	Malacostraca	Decapoda	Penaeidae
JR713575	Glp	Chinese mitten crab	*Callinectes sapidus*	Malacostraca	Decapoda	Varunidae
FE813068/FE813069	Glp	Flat porcelain crab	*Petrolisthes cinctipes*	Malacostraca	Decapoda	Porcellanidae
GAKD01001157	Glp	Amphipod	*Melita plumulosa*	Malacostraca	Amphipoda	Melitidae
GARO01026521	Glp	Amphipod	*Echinogammarus veneris*	Malacostraca	Amphipoda	Gammaridae
GAZX01003450	Glp 1	Caligid copepod	*Caligus rogercresseyi*	Maxillopoda	Siphonostomatoida	Caligidae
GAXK01070720	Glp 1a	Calanus	*Calanus finmarchicus*	Maxillopoda	Calanoida	Calanidae
GAXK01165253	Glp 1b	Calanus	*Calanus finmarchicus*	Maxillopoda	Calanoida	Calanidae
GAXK01179438	Glp 1c	Calanus	*Calanus finmarchicus*	Maxillopoda	Calanoida	Calanidae
KR005660	Glp 1_v1	Salmon louse	*Lepeophtheirus salmonis*	Maxillopoda	Siphonostomatoida	Caligidae
KR005661	Glp 1_v2	Salmon louse	*Lepeophtheirus salmonis*	Maxillopoda	Siphonostomatoida	Caligidae
JV199638	Glp 1a	Tide pool copepod	*Tigriopus californicus*	Maxillopoda	Harpacticoida	Harpacticidae
JV198720	Glp 1b	Tide pool copepod	*Tigriopus californicus*	Maxillopoda	Harpacticoida	Harpacticidae
GAZX01018815	Glp 3a	Caligid copepod	*Caligus rogercresseyi*	Maxillopoda	Siphonostomatoida	Caligidae
GAZX01001553	Glp 3b	Caligid copepod	*Caligus rogercresseyi*	Maxillopoda	Siphonostomatoida	Caligidae
GAXK01155600	Glp 3a	Calanus	*Calanus finmarchicus*	Maxillopoda	Calanoida	Calanidae
GAXK01155595	Glp 3b	Calanus	*Calanus finmarchicus*	Maxillopoda	Calanoida	Calanidae
KR005662	Glp 2	Salmon louse	*Lepeophtheirus salmonis*	Maxillopoda	Siphonostomatoida	Caligidae
KR005663	Glp 3_v1	Salmon louse	*Lepeophtheirus salmonis*	Maxillopoda	Siphonostomatoida	Caligidae
KR005664	Glp 3_v2	Salmon louse	*Lepeophtheirus salmonis*	Maxillopoda	Siphonostomatoida	Caligidae
JV199221	Glp 3a	Tide pool copepod	*Tigriopus californicus*	Maxillopoda	Harpacticoida	Harpacticidae
JV193385	Glp 3b	Tide pool copepod	*Tigriopus californicus*	Maxillopoda	Harpacticoida	Harpacticidae
GARW01015498	Glp 1	Cyclopid copepod	*Eucyclops serrulatus*	Maxillopoda	Cyclopoida	Cyclopidae
GARW01029074	Glp 3	Cyclopid copepod	*Eucyclops serrulatus*	Maxillopoda	Cyclopoida	Cyclopidae
EFX88758	Glp	Common water flea	*Daphnia pulex*	Branchiopoda	Diplostraca	Daphniidae
EFX88760	Glp	Common water flea	*Daphnia pulex*	Branchiopoda	Diplostraca	Daphniidae
EFX88757	Glp	Common water flea	*Daphnia pulex*	Branchiopoda	Diplostraca	Daphniidae
EFX66203	Glp	Common water flea	*Daphnia pulex*	Branchiopoda	Diplostraca	Daphniidae
EFX79826	Glp	Common water flea	*Daphnia pulex*	Branchiopoda	Diplostraca	Daphniidae
EFX88619	Glp	Common water flea	*Daphnia pulex*	Branchiopoda	Diplostraca	Daphniidae
BJ931504	Glp	Water flea	*Daphnia magna*	Branchiopoda	Diplostraca	Daphniidae
BJ935218/BJ935219	Glp	Water flea	*Daphnia magna*	Branchiopoda	Diplostraca	Daphniidae
GR508656	Glp	Water flea	*Daphnia carinata*	Branchiopoda	Diplostraca	Daphniidae
JW965215	Glp	Argulus	*Argulus siamensis*	Maxillopoda	Arguloida	Argulidae
JW969733	Glp	Argulus	*Argulus siamensis*	Maxillopoda	Arguloida	Argulidae
JW965635	Glp	Argulus	*Argulus siamensis*	Maxillopoda	Arguloida	Argulidae
JT035837	Glp A1	African social eresid spider	*Stegodyphus mimosarum*	Arachnida	Araneae	Eresidae
JT043093	Glp A1	African social eresid spider	*Stegodyphus tentoriicola*	Arachnida	Araneae	Eresidae
JT030770	Glp A1	African social eresid spider	*Stegodyphus lineatus*	Arachnida	Araneae	Eresidae
GBCS01013118	Glp A1	Western black widow	*Latrodectus hesperus*	Arachnida	Araneae	Theridiidae
AOMJ01012457/AOMJ01012461/AOMJ01099890	Glp A1	Common house spider	*Parasteatoda tepidariorum*	Arachnida	Araneae	Theridiidae
AYEL01063830/AYEL01063829/AYEL01055244	Glp A1	Chinese scorpion	*Mesobuthus martensii*	Arachnida	Scorpiones	Buthidae
AXZI01008059/AXZI01161692	Glp A1	Baja California bark scorpion	*Centruroides exilicauda*	Arachnida	Scorpiones	Buthidae
JT039102	Glp A2	African social eresid spider	*Stegodyphus mimosarum*	Arachnida	Araneae	Eresidae
JT042431	Glp A2	African social eresid spider	*Stegodyphus tentoriicola*	Arachnida	Araneae	Eresidae
JT030628	Glp A2	African social eresid spider	*Stegodyphus lineatus*	Arachnida	Araneae	Eresidae
GBCS01013464	Glp A2	Western black widow	*Latrodectus hesperus*	Arachnida	Araneae	Theridiidae
AOMJ01263225/AOMJ01263218	Glp A2	Common house spider	*Parasteatoda tepidariorum*	Arachnida	Araneae	Theridiidae
AYEL01089575/AYEL01071210	Glp A2	Chinese scorpion	*Mesobuthus martensii*	Arachnida	Scorpiones	Buthidae
JK732090	Glp A2	Brazilian scorpion	*Tityus serrulatus*	Arachnida	Scorpiones	Buthidae
AXZI01007911	Glp A2	Baja California bark scorpion	*Centruroides exilicauda*	Arachnida	Scorpiones	Buthidae
DR448116/DR447154	Glp A2L	São paulo black tarantula	*Acanthoscurria gomesiana*	Arachnida	Araneae	Theraphosidae
GAZS01013081	Glp A2L	Brazilian giant whiteknee tarantula	*Acanthoscurria geniculata*	Arachnida	Araneae	Theraphosidae
AEO32269	Glp A	Gulf coast tick	*Amblyomma maculatum*	Acari	Ixodida	Ixodidae
JAC33429	Glp A	Rickettsia tick	*Amblyomma triste*	Acari	Ixodida	Ixodidae
JAC25831	Glp A	Rickettsia tick	*Amblyomma parvum*	Acari	Ixodida	Ixodidae
JAC21331	Glp A	Cayenne tick	*Amblyomma cajennense*	Acari	Ixodida	Ixodidae
GACK01004617	Glp A	Zebra tick	*Rhipicephalus pulchellus*	Acari	Ixodida	Ixodidae
CD794748/CD794747	Glp A	Brown ear tick	*Rhipicephalus appendiculatus*	Acari	Ixodida	Ixodidae
CAX48963	Glp A	Brown dog tick	*Rhipicephalus sanguineus*	Acari	Ixodida	Ixodidae
JAA73021	Glp A	Castor bean tick	*Ixodes ricinus*	Acari	Ixodida	Ixodidae
ABJB010692148/ABJB010720745/ABJB010408716/ ABJB010406759	Glp A	Blacklegged tick	*Ixodes scapularis*	Acari	Ixodida	Ixodidae
XP_003745281	Glp A	Western predatory mite	*Metaseiulus occidentalis*	Acari	Mesostigmata	Phytoseiidae
JL016378	Glp A	Western predatory mite	*Metaseiulus occidentalis*	Acari	Mesostigmata	Phytoseiidae
CAEY01000889	Glp A	Two-spotted spider mite	*Tetranychus urticae*	Acari	Eleutherengona	Tetranychidae
tetur04g02680	Glp A	Two-spotted spider mite	*Tetranychus urticae*	Acari	Eleutherengona	Tetranychidae
tetur01g15060	Glp A	Two-spotted spider mite	*Tetranychus urticae*	Acari	Eleutherengona	Tetranychidae
AEO36644	Glp B1	Gulf coast tick	*Amblyomma maculatum*	Acari	Ixodida	Ixodidae

JAC35177	Glp B1	Rickettsia tick	*Amblyomma triste*	Acari	Ixodida	Ixodidae
JAC25752	Glp B1	Rickettsia tick	*Amblyomma parvum*	Acari	Ixodida	Ixodidae
JAC22662	Glp B1	Cayenne tick	*Amblyomma cajennense*	Acari	Ixodida	Ixodidae
GACK01008102	Glp B1	Zebra tick	*Rhipicephalus pulchellus*	Acari	Ixodida	Ixodidae
CAR66115	Glp B1	Brown dog tick	*Rhipicephalus sanguineus*	Acari	Ixodida	Ixodidae
CD780384	Glp B1	Brown ear tick	*Rhipicephalus appendiculatus*	Acari	Ixodida	Ixodidae
ABJB010086453/ABJB010131197/ABJB010002103	Glp B2a	Blacklegged tick	*Ixodes scapularis*	Acari	Ixodida	Ixodidae
EEC04800	Glp B2b	Blacklegged tick	*Ixodes scapularis*	Acari	Ixodida	Ixodidae
CAX48964	Glp B2a	Castor bean tick	*Ixodes ricinus*	Acari	Ixodida	Ixodidae
JAA71533	Glp B2b	Castor bean tick	*Ixodes ricinus*	Acari	Ixodida	Ixodidae
JAC32051	Glp B2	Rickettsia tick	*Amblyomma triste*	Acari	Ixodida	Ixodidae
JAC25679	Glp B2	Rickettsia tick	*Amblyomma parvum*	Acari	Ixodida	Ixodidae
JAC19979	Glp B2	Cayenne tick	*Amblyomma cajennense*	Acari	Ixodida	Ixodidae
JAC25782	Glp B3	Rickettsia tick	*Amblyomma parvum*	Acari	Ixodida	Ixodidae
JAC21377	Glp B3	Cayenne tick	*Amblyomma cajennense*	Acari	Ixodida	Ixodidae
GAGD01000711	Glp B3	Lone Star tick	*Amblyomma americanum*	Acari	Ixodida	Ixodidae
JAA56054	Glp B3	Zebra tick	*Rhipicephalus pulchellus*	Acari	Ixodida	Ixodidae
GADI01002277	Glp B3	Castor bean tick	*Ixodes ricinus*	Acari	Ixodida	Ixodidae
EEC20079	Glp B3	Blacklegged tick	*Ixodes scapularis*	Acari	Ixodida	Ixodidae
ABI53034	Glp B4	American dog tick	*Dermacentor variabilis*	Acari	Ixodida	Ixodidae
CK190314/CK190314	Glp B4	Southern cattle tick	*Rhipicephalus microplus*	Acari	Ixodida	Ixodidae
ABJB011072005/ABJB010219880	Glp B4	Blacklegged tick	*Ixodes scapularis*	Acari	Ixodida	Ixodidae
GANL01004539	Glp B	Black widow	*Latrodectus tredecimguttatus*	Arachnida	Araneae	Theridiidae
GBCS01000276	Glp B	Western black widow	*Latrodectus hesperus*	Arachnida	Araneae	Theridiidae
AOMJ01123248/AOMJ01123250/AOMJ01123251	Glp B	Common house spider	*Parasteatoda tepidariorum*	Arachnida	Araneae	Theridiidae
AOMJ01110087/AOMJ01110082/AOMJ01110081/AOMJ01110079	Glp B	Common house spider	*Parasteatoda tepidariorum*	Arachnida	Araneae	Theridiidae
GAZS01045747	Glp B	Brazilian giant whiteknee tarantula	*Acanthoscurria geniculata*	Arachnida	Araneae	Theraphosidae
tetur01g07330	Glp B	Two-spotted spider mite	*Tetranychus urticae*	Acari	Eleutherengona	Tetranychidae
CAEY01001013/CAEY01001014	Glp B	Two-spotted spider mite	*Tetranychus urticae*	Acari	Eleutherengona	Tetranychidae
CAEY01001579	Glp B	Two-spotted spider mite	*Tetranychus urticae*	Acari	Eleutherengona	Tetranychidae
JT037994	Glp B	African social eresid spider	*Stegodyphus mimosarum*	Arachnida	Araneae	Eresidae
JT030404	Glp B	African social eresid spider	*Stegodyphus lineatus*	Arachnida	Araneae	Eresidae
GBCS01013198	Glp B	Western black widow	*Latrodectus hesperus*	Arachnida	Araneae	Theridiidae
AYEL01071210	Glp B	Chinese scorpion	*Mesobuthus martensii*	Arachnida	Scorpiones	Buthidae
AXZI01007912/AXZI01007914/AXZI01007916	Glp B	Baja California bark scorpion	*Centruroides exilicauda*	Arachnida	Scorpiones	Buthidae

Other Protosotomia aquaglyceroporins

AEP14563	Glp 9	Water bear	*Milnesium tardigradum*	Tardigrada	Apochela	Milnesiidae
AEP14555	Glp (1)	Water bear	*Milnesium tardigradum*	Tardigrada	Apochela	Milnesiidae
AEP14558	Glp (4)	Water bear	*Milnesium tardigradum*	Tardigrada	Apochela	Milnesiidae
AEP14562	Glp (8)	Water bear	*Milnesium tardigradum*	Tardigrada	Apochela	Milnesiidae
AEP14556	Glp (2)	Water bear	*Milnesium tardigradum*	Tardigrada	Apochela	Milnesiidae
AEP14557	Glp (3)	Water bear	*Milnesium tardigradum*	Tardigrada	Apochela	Milnesiidae
AEP14564	Glp (10)	Water bear	*Milnesium tardigradum*	Tardigrada	Apochela	Milnesiidae
AEP14561	Glp (7)	Water bear	*Milnesium tardigradum*	Tardigrada	Apochela	Milnesiidae

Arthropoda unorthodox aquaporins

FBpp0086879	Aqp 12L	Fruit fly	*Drosophila melanogaster*	Hexapoda	Diptera	Drosophilidae
FBpp0201777	Aqp 12L	Fruit fly	*Drosophila sechellia*	Hexapoda	Diptera	Drosophilidae
FBpp0258396	Aqp 12L	Fruit fly	*Drosophila yakuba*	Hexapoda	Diptera	Drosophilidae
FBpp0141060	Aqp 12L	Fruit fly	*Drosophila erecta*	Hexapoda	Diptera	Drosophilidae
FBpp0224314	Aqp 12L	Fruit fly	*Drosophila simulans*	Hexapoda	Diptera	Drosophilidae
FBpp0116155	Aqp 12L	Fruit fly	*Drosophila ananassae*	Hexapoda	Diptera	Drosophilidae
FBpp0276666	Aqp 12L	Fruit fly	*Drosophila pseudoobscura*	Hexapoda	Diptera	Drosophilidae
FBpp0235888	Aqp 12L	Fruit fly	*Drosophila virilis*	Hexapoda	Diptera	Drosophilidae
FBpp0167611	Aqp 12L	Fruit fly	*Drosophila mojavensis*	Hexapoda	Diptera	Drosophilidae
FBpp0158826	Aqp 12L	Fruit fly	*Drosophila grimshawi*	Hexapoda	Diptera	Drosophilidae
FBpp0247034	Aqp 12L	Fruit fly	*Drosophila willistoni*	Hexapoda	Diptera	Drosophilidae
GAKB01003759	Aqp 12L	Olive fruit fly	*Bactrocera oleae*	Hexapoda	Diptera	Tephritidae
XP_004529905	Aqp 12L	Mediterranean fruit fly	*Ceratitis capitata*	Hexapoda	Diptera	Tephritidae
CAQQ02174372/CAQQ02174371/CAQQ02389937	Aqp 12L	Scuttle fly	*Megaselia scalaris*	Hexapoda	Diptera	Phoridae
EZ602043	Aqp 12L	Flesh fly	*Sarcophaga crassipalpis*	Hexapoda	Diptera	Sarcophagidae
FD463879	Aqp 12L	Horn fly	*Haematobia irritans irritans*	Hexapoda	Diptera	Muscidae
AQPM01092757	Aqp 12L	House fly	*Haematobia irritans irritans*	Hexapoda	Diptera	Muscidae
AFP49900	Aqp 12L	Tsetse fly	*Glossina morsitans morsitans*	Hexapoda	Diptera	Glossinidae
AAEL014255/XP_001648046	Aqp 12L	Yellow fever mosquito	*Aedes aegypti*	Hexapoda	Diptera	Culicidae
CPIJ004456	Aqp 12L	Southern house mosquito	*Culex quinquefasciatus*	Hexapoda	Diptera	Culicidae
AGAP010878	Aqp 12L	African malaria mosquito	*Anopheles gambiae*	Hexapoda	Diptera	Culicidae
EZ978777	Aqp 12L	African malaria mosquito	*Anopheles funestus*	Hexapoda	Diptera	Culicidae
EFR26850	Aqp 12L	American malaria mosquito	*Anopheles darlingi*	Hexapoda	Diptera	Culicidae
GAFE01006687	Aqp 12L	Indonesian malaria mosquito	*Anopheles sinensis*	Hexapoda	Diptera	Culicidae
GAAK01006945	Aqp 12L	Antarctic flightless midge	*Belgica antarctica*	Hexapoda	Diptera	Chironomidae
AEGA01009001/AEGA01031906	Aqp 12L	Hessian fly	*Mayetiola destructor*	Hexapoda	Diptera	Cecidomyiidae
JP551754	Aqp 12L	Sand fly	*Phlebotomus papatasi*	Hexapoda	Diptera	Psychodidae
AM094572/AM094570	Aqp 12L	Sand fly	*Lutzomyia longipalpis*	Hexapoda	Diptera	Psychodidae
HX266982	Aqp 12L	Domestic silkworm	*Bombyx mori*	Hexapoda	Lepidoptera	Bombycidae
CAEZ01010288	Aqp 12L	Postman butterfly	*Heliconius melpomene melpomene*	Hexapoda	Lepidoptera	Nymphalidae
EHJ69710	Aqp 12L	Monarch butterfly	*Danaus plexippus*	Hexapoda	Lepidoptera	Nymphalidae
GAFU01002466	Aqp 12L	Beet armyworm	*Spodoptera exigua*	Hexapoda	Lepidoptera	Noctuidae
GT199640/GT196938	Aqp 12L	Tobacco budworm	*Heliothis virescens*	Hexapoda	Lepidoptera	Noctuidae
HS099242	Aqp 12L	Maruca pod borer	*Maruca vitrata*	Hexapoda	Lepidoptera	Crambidae
JP615554/JP615553	Aqp 12L	Propertius duskywing	*Erynnis propertius*	Hexapoda	Lepidoptera	Hesperiidae
AIXA01009041	Aqp 12L	Tobacco hornworm	*Manduca sexta*	Hexapoda	Lepidoptera	Sphingidae
HS099242	Aqp 12L	Maruca pod borer	*Maruca vitrata*	Hexapoda	Lepidoptera	Crambidae
BAGR01006886/BAGR01078921/BAGR01078920	Aqp 12L	Diamondback Moth	*Plutella xylostella*	Hexapoda	Lepidoptera	Plutellidae
GAXW01087864	Aqp 12L	Antlion	*Euroleon nostras*	Hexapoda	Neoptera	Myrmeleontidae
GAVV01020125	Aqp 12L	Green lacewing	*Pseudomallada prasinus*	Hexapoda	Neoptera	Chrysopidae
XP_974208	Aqp 12L	Red flour Beetle	*Tribolium castaneum*	Hexapoda	Coleoptera	Tenebrionidae
AQHT01000891/AQHT01000892	Aqp 12L	Asian longhorned beetle	*Anoplophora glabripennis*	Hexapoda	Coleoptera	Cerambycidae
GAFI01011142	Aqp 12L	Southern pine beetle	*Dendroctonus frontalis*	Hexapoda	Coleoptera	Curculionidae
APGL01029398	Aqp 12L	Mountain pine beetle	*Dendroctonus ponderosae*	Hexapoda	Coleoptera	Curculionidae
JR469890	Aqp 12L	Red palm weevil	*Rhynchophorus ferrugineus*	Hexapoda	Coleoptera	Curculionidae
GAEO01001684	Aqp 12L	White pine weevil	*Pissodes strobi*	Hexapoda	Coleoptera	Curculionidae
AGRH01000202	Aqp 12L	Reticulated beetle	*Priacma serrata*	Hexapoda	Coleoptera	Cupedidae
XP_003393649	Aqp 12L	Buff-tailed bumblebee	*Bombus terrestris*	Hexapoda	Hymenoptera	Apidae
XP_003490105	Aqp 12L	Common eastern bumble bee	*Bombus impatiens*	Hexapoda	Hymenoptera	Apidae
XP_001119893	Aqp 12L	Honey bee	*Apis mellifera*	Hexapoda	Hymenoptera	Apidae
ANOB01024207	Aqp 12L	Halictid Bee	*Lasioglossum albipes*	Hexapoda	Hymenoptera	Halictidae
GAGH01010151	Aqp 12L	Mason bee	*Osmia cornuta*	Hexapoda	Hymenoptera	Megachilidae
AFJA01002269	Aqp 12L	Alfalfa leafcutting bee	*Megachile rotundata*	Hexapoda	Hymenoptera	Megachilidae

EFN85024	Aqp 12L	Jerdon's jumping ant	*Harpegnathos saltator*	Hexapoda	Hymenoptera	Formicidae
EFN70241	Aqp 12L	Florida carpenter ant	*Camponotus floridanus*	Hexapoda	Hymenoptera	Formicidae
ADTU01018182	Aqp 12L	Leafcutter ant	*Atta cephalotes*	Hexapoda	Hymenoptera	Formicidae
EGI57993	Aqp 12L	Panamanian leafcutter ant	*Acromyrmex echinatior*	Hexapoda	Hymenoptera	Formicidae
FQ836707/FQ840903	Aqp 12L	Parasitoid wasp	*Asobara tabida*	Hexapoda	Hymenoptera	Braconidae
AOFN01007317	Aqp 12L	Coleseed sawfly	*Athalia rosae*	Hexapoda	Hymenoptera	Tenthredinidae
PHUM127940	Aqp 12L	Human body louse	*Pediculus humanus corporis*	Hexapoda	Phthiraptera	Pediculidae
ACPB02038779/ACPB02002207	Aqp 12L	Assassin bug	*Rhodnius prolixus*	Hexapoda	Hemiptera	Reduviidae
KF048101	Aqp 12L	Lygus bug	*Lygus hesperus*	Hexapoda	Hemiptera	Miridae
EZ940769/HP803079	Aqp 12L	Sweet potato whitefly	*Bemisia tabaci*	Hexapoda	Hemiptera	Aleyrodidae
HS430594	Aqp 12L	Brown planthopper	*Nilaparvata lugens*	Hexapoda	Hemiptera	Delphacidae
GAGF01017370	Aqp 12L	Green lacewing	*Chrysopa pallens*	Hexapoda	Neoptera	Chrysopidae
FG130040	Aqp 12L	American cockroach	*Periplaneta americana*	Hexapoda	Blattodea	Blattidae
GBID01003279	Aqp 12L	German cockroach	*Blattella germanica*	Hexapoda	Blattodea	Ectobiidae
AUST01032598/AUST01032597	Aqp 12L	Nevada dampwood termite	*Zootermopsis nevadensis*	Hexapoda	Isoptera	Termopsidae
FX376324	Aqp 12L	Wood-eating higher termite	*Nasutitermes takasagoensis*	Hexapoda	Isoptera	Termitidae
GABA01001239	Aqp 12L	Zoraptid	*Zorotypus gurneyi*	Hexapoda	Zoraptera	Zorotypidae
GAWE01136841/GAWE01162972/GAWE01022485	Aqp 12L	Vietnamese walking stick insect	*Ramulus artemis*	Hexapoda	Phasmatodea	Phasmatidae
GAIZ01025748	Aqp 12L	Sand field cricket	*Gryllus firmus*	Hexapoda	Orthoptera	Gryllidae
EH632839	Aqp 12L	Hawaian swordtail cricket	*Laupala kohalensis*	Hexapoda	Orthoptera	Gryllidae
CO854318/CO854607	Aqp 12L	Migratory locust	*Locusta migratoria*	Hexapoda	Orthoptera	Acrididae
GAEQ01010065	Aqp 12L	Hagen´s bluet	*Enallagma hageni*	Hexapoda	Odonata	Coenagrionidae
APVN01144267	Aqp 12L	Scarce chaser	*Ladona fulva*	Hexapoda	Odonata	Libellulidae
AYNC01076740	Aqp 12L	Green drake	*Ephemera danica*	Hexapoda	Ephemeroptera	Ephemeridae
GAMM01011672	Aqp 12L	Springtail	*Orchesella cincta*	Hexapoda	Collembola	Entomobryidae
GAMN01009381	Aqp 12L	Springtail	*Folsomia candida*	Hexapoda	Collembola	Isotomidae
AFFK01022088	Aqp 12L	Coastal European centipede	*Strigamia maritima*	Chilopoda	Geophilomorpha	Linotaeniidae
JR223268	Aqp 12L	Black tiger shrimp	*Penaeus monodon*	Malacostraca	Decapoda	Penaeidae
JP419814/JP406388	Aqp 12L	Pacific white shrimp	*Litopenaeus vannamei*	Malacostraca	Decapoda	Penaeidae
EX568605	Aqp 12L	American lobster	*Homarus americanus*	Malacostraca	Decapoda	Nephropidae
FE773872	Aqp 12L	Flat porcelain crab	*Petrolisthes cinctipes*	Malacostraca	Decapoda	Porcellanidae
DN634964	Aqp 12L	Green shore Crab	*Carcinus maenas*	Malacostraca	Decapoda	Carcinidae
GAKD01002926	Aqp 12L	Amphipod	*Melita plumulosa*	Malacostraca	Amphipoda	Melitidae
ES505590	Aqp 12L	Brine Shrimp	*Artemia franciscana*	Branchiopoda	Anostraca	Artemiidae
EFX67799	Aqp 12L	Common water flea	*Daphnia pulex*	Branchiopoda	Diplostraca	Daphniidae
ACO11400	Aqp 12L	Caligid copepod	*Caligus rogercresseyi*	Maxillopoda	Siphonostomatoida	Caligidae
ACO14967	Aqp 12L	Caligid copepod	*Caligus clemensi*	Maxillopoda	Siphonostomatoida	Caligidae
KR005665	Aqp 12L1	Salmon louse	*Lepeophtheirus salmonis*	Maxillopoda	Siphonostomatoida	Caligidae
KR005666	Aqp 12L2	Salmon louse	*Lepeophtheirus salmonis*	Maxillopoda	Siphonostomatoida	Caligidae
GO412603	Aqp 12L	Cod worm	*Lernaeocera branchialis*	Maxillopoda	Siphonostomatoida	Pennellidae
EH666294	Aqp 12L	Calanus	*Calanus finmarchicus*	Maxillopoda	Calanoida	Calanidae
XP_002416580	Aqp 12L	Blacklegged tick	*Ixodes scapularis*	Acari	Ixodida	Ixodidae
ADDG01005800	Aqp 12L	Honeybee mite	*Varroa destructor*	Acari	Mesostigmata	Varroidae
XP_003740565	Aqp 12L	Western predatory mite	*Metaseiulus occidentalis*	Acari	Mesostigmata	Phytoseiidae
JR698214	Aqp 12L	Two-spotted spider mite	*Tetranychus urticae*	Acari	Acariformes	Tetranychidae
GANL01000406	Aqp 12L	Black widow	*Latrodectus tredecimguttatus*	Arachnida	Araneae	Theridiidae
GBCS01016346	Aqp 12L	Western black widow	*Latrodectus hesperus*	Arachnida	Araneae	Theridiidae
AOMJ01188201/AOMJ01188200/AOMJ01188199	Aqp 12L	Common house spider	*Parasteatoda tepidariorum*	Arachnida	Araneae	Theridiidae
JT033535	Aqp 12L	Subsocial spider	*Stegodyphus lineatus*	Arachnida	Araneae	Theridiidae
EY189672	Aqp 12L	Spider	*Loxosceles laeta*	Arachnida	Araneae	Sicariidae
GAZS01072938	Aqp 12L	Brazilian giant whiteknee tarantula	*Acanthoscurria geniculata*	Arachnida	Araneae	Theraphosidae
AYEL01083560	Aqp 12L	Chinese scorpion	*Mesobuthus martensii*	Arachnida	Scorpiones	Buthidae
AXZI01164565/AXZI01164567	Aqp 12L	Baja California bark scorpion	*Centruroides exilicauda*	Arachnida	Scorpiones	Buthidae
AEP14565	Aqp 12L(11)	Water bear	*Milnesium tardigradum*	Tardigrada	Apochela	Milnesiidae
**Bacteria AqpZ**						
AAC43518/EBESCP00000004569	Aqp Z	Bacteria	*Escherichia coli*	Proteobacteria	Enterobacteriales	Enterobacteriaceae
WP_005067324/EBESCP00000088383	Aqp Z	Bacteria	*Shigella flexneri*	Proteobacteria	Enterobacteriales	Enterobacteriaceae
YP_038558/EBBACP00000072619	Aqp Z	Bacteria	*Bacillus thuringiensis*	Firmicutes	Bacillales	Bacillaceae
YP_002340560/EBBACP00000092199	Aqp Z	Bacteria	*Bacillus cereus*	Firmicutes	Bacillales	Bacillaceae
NP_359196/EBSTRP00000015353	Aqp Z	Bacteria	*Streptococcus pneumoniae*	Firmicutes	Lactobacillales	Streptococcaceae
**Bacteria GlpF**						
NP_290556/EBESCP00000004421	Glp F	Bacteria	*Escherichia coli*	Proteobacteria	Enterobacteriales	Enterobacteriaceae
WP_000084271/EBESCP00000089272	Glp F	Bacteria	*Shigella flexneri*	Proteobacteria	Enterobacteriales	Enterobacteriaceae
YP_405253/EBESCP00000079777	Glp F	Bacteria	*Shigella dysenteriae*	Proteobacteria	Enterobacteriales	Enterobacteriaceae
YP_003615521	Glp F	Bacteria	*Enterobacter cloacae subsp.*	Proteobacteria	Enterobacteriales	Enterobacteriaceae
WP_010299311	Glp F	Bacteria	*Pectobacterium carotovorum*	Proteobacteria	Enterobacteriales	Enterobacteriaceae
NP_977447/EBBACP00000096005	Glp F	Bacteria	*Bacillus cereus*	Firmicutes	Bacillales	Bacillaceae
YP_035285/EBBACP00000073529	Glp F	Bacteria	*Bacillus thuringiensis*	Firmicutes	Bacillales	Bacillaceae
NP_388809/EBBACP00000003509	Glp F	Bacteria	*Bacillus subtilis subsp.*	Firmicutes	Bacillales	Bacillaceae
NP_359579/EBSTRP00000014534	Glp F3	Bacteria	*Streptococcus pneumoniae*	Firmicutes	Lactobacillales	Streptococcaceae
**Hexapod bacteroid (endosymbiont) GlpF**						
XM_004532995	Glp F	Mediterranean fruit fly	*Ceratitis capitata*	Hexapoda	Diptera	Tephritidae
ACPB02009318	Glp F	Assassin bug	*Rhodnius prolixus*	Hexapoda	Hemiptera	Reduviidae
**Archaea AqpM**						
AB055880	Aqp M	Archaea	*Methanothermobacter thermautotrophicus*	Euryarchaeota	Methanobacteriales	Methanobacteriaceae
NP_988083	Aqp M	Archaea	*Methanococcus maripaludis*	Euryarchaeota	Methanococcales	Methanococcaceae
YP_003435795	Aqp M	Archaea	*Ferroglobus placidus*	Euryarchaeota	Archaeoglobales	Archaeoglobaceae
YP_843562	Aqp M	Archaea	*Methanosaeta thermophila*	Euryarchaeota	Methanosarcinales	Methanosaetaceae
YP_305556	Aqp M	Archaea	*Methanosarcina barkeri*	Euryarchaeota	Methanosarcinales	Methanosarcinaceae
